# Design of stapled peptide-based PROTACs for MDM2/MDMX atypical degradation and tumor suppression

**DOI:** 10.7150/thno.75444

**Published:** 2022-09-11

**Authors:** Si Chen, Xiang Li, Yinghua Li, Xing Yuan, Chenchen Geng, Songyan Gao, Jinyang Li, Bohan Ma, Zhe Wang, Wuyuan Lu, Hong-Gang Hu

**Affiliations:** 1 School of Medicine or Institute of Translational Medicine, Shanghai University, Shanghai 200444, China.; 2 School of Pharmacy, Second Military Medical University, Shanghai 200433, China.; 3 Key Laboratory of Medical Molecular Virology (MOE/NHC/CAMS), School of Basic Medical Sciences, Fudan University, Shanghai 200030, China.; 4 The First Affiliated Hospital of Xi'an Jiaotong University, Xi'an, 710049, China.; 5 Institute of Bioengineering, College of Chemical and Biological Engineering, Zhejiang University, Hangzhou, Zhejiang 310027, China.

**Keywords:** MDM2/MDMX, p53, stapled peptide, PROTAC, anticancer

## Abstract

**Rationale:** Although stapled peptides offer a powerful solution to overcome the susceptibility of linear peptides to proteolytic degradation and improve their ability to cross membranes, an efficient and durable disease treatment strategy has not yet been developed due to the inevitable elimination of peptide inhibitors and rapid accumulation of target proteins.

**Methods:** Herein we developed stapled peptide-based proteolysis-targeting chimeras (SP-PROTACs), that simultaneously exhibited improved cellular uptake and proteolytic stability attributed to the stapled peptides, and efficient target protein degradation promoted by the PROTACs. Based on the PMI peptide with dual specificity for both MDM2 and MDMX, a series of SP-PROTACs were designed.

**Results:** Among them, the optimized SPMI-HIF2-1 exhibited similar binding affinity with MDM2 and MDMX but obviously higher helical contents, improved proteolytic stability, better cellular permeability, and a better pharmacokinetic profile compared with its linear counterpart. Importantly, SPMI-HIF2-1 could effectively kill cancer cells and inhibit tumor progression in subcutaneous and orthotopic colorectal cancer xenograft models through simultaneously promoting the atypical degradation of both MDM2 and MDMX and durable p53 activation. An FP-based binding assay and structural modeling analysis of the ternary complex suggested that SPMI-HIF2-1 simultaneously bound with the target protein and E3 ligase.

**Conclusion:** Our findings not only provide a new class of anticancer drug candidates, but also bridge the gap and reduce the physical distance between peptides and PROTACs.

## Introduction

Approximately one-third of human tumors are related to p53 inhibition and its intracellular degradation. The tumor suppressor protein p53 is negatively regulated by the oncogenic proteins murine double minute 2 (MDM2) and murine double minute X (MDMX) 1-3. Therefore, antagonizing both MDM2 and MDMX to activate and stabilize p53 is an important strategy for antitumor drug design 4-7. Small molecule antagonists that inhibit the interactions between p53 and MDM2 or MDMX are likely to cause “off-target” toxicity due to poor binding affinity and specificity. Some notable examples, including spiro-oxindole-derived compounds and the *cis*-imidazoline analog Nutlin-3, have recently failed to pass clinical trials 8-10.

Peptides are superior to small molecules in inhibiting p53-MDM2/MDMX interactions because peptides can antagonize both MDM2 and MDMX at high affinities 11-14. A growing body of evidence indicates that MDM2 and MDMX cooperatively inhibit p53 activity and reduce its cellular stability in some tumors, and that dual-specificity antagonists are needed to achieve robust and sustained p53 activation for optimal therapeutic efficacy. Peptide inhibition of the interactions between p53 and its negative regulators MDM2 and MDMX could activate p53 both *in vitro* and *in vivo*, representing a viable and potent therapeutic strategy for cancer treatment. However, linear peptides suffer from proteolytic degradation and a low ability to cross membranes.

Stapled peptides, which are characterized by a covalent bond located at a peptide side chain, exhibit better cellular uptake and proteolytic stability than their linear counterparts and have therefore attracted increasing attention in peptide drug discovery 15, 16. Targeting p53-MDM2/MDMX interactions, a series of stapled peptides originating from different stapling chemistries have been designed, of which the notable example ALRN-6924, a hydrocarbon stapled MDM2/MDMX antagonist, can reactivate the p53 pathway, and this drug candidate has entered phase II clinical trials for advanced solid tumors and lymphomas 17.

In a previous study, a potent dodecameric peptide antagonist (TS**F**AE**YW**AL**L**SP), termed PMI-N8A, with dual specificity for both MDM2 and MDMX was identified using phage display techniques and systematic mutational analysis 12, 13. Structural studies of PMI and PMI-N8A in complex with both MDM2 and MDMX indicated four critical hydrophobic residues for energetic contributions: Phe3, Tyr6, Trp7, and Leu10.^12^, ^13^ Modeled after the PMI-N8A peptide, the hydrocarbon stapled PMI peptides were designed with very strong and persistent p53-activating activity *in vitro*, which surpassed Nutlin-3 in potency but displayed significantly lower toxicity 18. However, it still remained challenging to design stapled PMI peptides that efficiently inhibit p53-dependent tumor growth in cell culture and, more importantly, *in vivo*, due in part to the rapid accumulation of both MDM2 and MDMX in cancer cells. To overcome the potential limitations of MDM2/MDMX antagonists, novel strategies are urgently needed to achieve robust and sustained degradation of MDM2 and MDMX and tumor suppression.

Proteolysis-targeting chimeras (PROTACs), which consist of two specific ligands for the intracellular target protein and E3 ubiquitin ligase, respectively, have now emerged as a powerful bifunctional tool to efficiently degrade proteins of interest 19-27. Peptide-based PROTACs can be more rationally and easily designed than the conventional small molecule-based ones by utilizing protein or peptide motifs identified from protein-protein interaction (PPI) partners, especially in the “undruggable” PPIs 28. Furthermore, stapled peptide-based PROTACs (SP-PROTACs) combine the advantages of high binding affinity and effective protein elimination, thereby representing a novel approach for PROTAC design 15. As a proof of concept, we previously designed the first-generation potent β-catenin degrader, which exhibited excellent tumor prevention and cure ability in cell culture and *in vivo*, highlighting the potential of SP-PROTACs to be developed into peptide drugs 29.

Von Hippel Lindau (VHL) factor is a component of the pentameric Cullin RING E3 ubiquitin ligase (CRL2^VHL^) complex 30-32 and its effective recruitment has already been applied in peptide-based PROTAC design 28, 29, 33. Herein with the aid of VHL binding peptide, we designed a series of SPMI-based SP-PROTACs using different stapling positions and spacer linkers. Among them, the optimized SPMI-HIF2-1 showed significantly stronger inhibition of colorectal cancer (CRC) cells than the linear peptide PMI-HIF1-1 and its stapled counterpart SPMI2. SPMI-HIF2-1 displayed excellent cancer cell specificity between HCT116 p53^+/+^ and HCT116 p53^-/-^. Of note, SPMI-HIF2-1 could increase the protein levels of both MDM2 and MDMX at concentrations below 20 μM and resulted in degradation of target proteins in a dose-dependent manner at concentrations ranging from 20 to 100 μM. The above results, combined with western blot, apoptosis, and cell cycle analyses, indicate that SPMI-HIF2-1 could kill cancer cells through a simultaneous atypical degradation of both MDM2 and MDMX and durable p53 activation. SPMI-HIF2-1 exhibited similar binding affinity with MDM2 and MDMX, but obviously higher helical contents, enhanced proteolytic stability, and higher cellular uptake compared with its linear counterpart. Furthermore, SPMI-HIF2-1 could effectively inhibit tumor progression in subcutaneous and intrarectal nude mouse xenograft models, through a simultaneous degradation of both MDM2 and MDMX and durable p53 activation. Pharmacokinetic analysis revealed that SPMI-HIF2-1 was similar to SPMI2, but with obviously better parameters, including area under the curve (AUC), mean residence time (MRT), and half-time values, than PMI-HIF1-1. A fluorescence polarization (FP)-based binding assay and structural modeling analysis of ternary complexes between SPMI-HIF2-1, MDM2 or MDMX, and VHL suggested that this optimized SP-PROTAC could simultaneously target both MDM2 and VHL, similar to the binary binding, thereby facilitating the interaction between the target protein and E3 ligase for the subsequent ubiquitination-dependent degradation process.

## Methods

### SP-PROTAC synthesis

All reagents and solvents used during the experiments were purchased from Titan Scientific Co., Ltd. Rink Amide MBHA resin was obtained from Tianjin Nankai Hecheng Science & Technology Co., Ltd. Rink amide MBHA resin was used as the solid phase support, and SPPS was used for the assembly of the linear peptide sequence. First, resin was soaked in dichloromethane (DCM) and dimethyl formamide (DMF) for 20 min and filtered. Fmoc deprotection was performed with piperidine (20% in DMF) for 5 min twice. Then Fmoc-AA-OH (1 mmol, 10 equivalents), Oxyma (1 mmol, 10 equivalents), DIC (2 mmol, 20 equivalents), and NMP (6 ml) were mixed, added to the resin, and shaken for 20 min at 60 °C to complete the coupling of the first amino acid. The peptide couplings of Fmoc-S5-OH and Fmoc-R8-OH were carried out over a single 2-h coupling cycle using two equivalents of the Fmoc protected amino acids at 60 °C. RCM reactions were performed through the addition of first-generation Grubbs' catalyst (20 mol% in DCM) for 2 h, which was repeated to ensure a complete reaction. After the RCM reaction was complete, a test cleavage was performed to ensure adequate yield. The target peptides were cleaved from the resin, purified by RP-HPLC, and verified by HR-MS.

### Cell culture and animal maintenance

The human colon carcinoma cell line HCT116 p53^+/+^ was obtained from the Cell Bank of the Chinese Academy of Sciences (Shanghai, China). HCT116 p53^-/-^ cells were generously provided by Prof. Vogelstein of Johns Hopkins University. Both cell lines were cultured in McCoy's 5A medium (Gibco, USA) containing 10% FBS, 100 units/ml penicillin, and 100 μg/ml streptomycin at 37 °C in a humidified incubator containing 5% CO_2_.

Male BALB/c nude mice (15-20 g) were purchased from Shanghai Xipuer-Bikai Laboratory Animal Co., Ltd. (Shanghai, China). The animals were maintained under specific pathogen-free conditions with food and water supplied ad libitum in the Laboratory Animal Center of the Naval Medical University. All animal experiments were carried out in accordance with the Guide for the Care and Use of Laboratory Animals of the National Institutes of Health and were approved by the Committee on the Ethics of Animal Experiments of the Naval Military Medical University, China.

### Cell viability assay

Cell viability was measured using a Cell Counting Kit-8 (CCK-8) (Dojindo, Kumamoto, Japan) according to the manufacturer's instructions. In brief, the cells (5 × 10^3^ cells/well in 100 ml medium) were cultured for 24 h in 96-well microplates in a humidified 5% CO_2_ incubator at 37 °C and then treated with varying concentrations of compounds or 0.1% DMSO vehicle control. After incubation for 72 h, 10 μl of CCK8 solution was added to each well, and then the samples were incubated for an additional 1 h before the absorbance was measured at 450 nm by a Cytation5 Microplate reader (BioTek).

### Western blot analysis

HCT116, MCF-7, or other cells were seeded in six-well plates (4 × 10^5^ cells/well) and treated with PROTACs at the indicated concentrations for 24 h. The cells were lysed in RIPA lysis buffer. The protein concentrations were measured using the BCA method and equal amounts of each sample were separated by SDS-PAGE, followed by transfer onto filter membranes. Then, the membranes were blocked and incubated with primary antibodies (Abcam, UK) at 4 °C overnight, followed by incubation with the secondary antibodies. The protein bands were identified using a ChemiDoc MP Imaging System (Bio-Rad, USA).

### Real-time quantitative PCR

First, 2 × 10^5^ HCT116 or MCF-7 cells were seeded in 12-well plates and treated with peptides at various concentrations for 24 h. Total RNA was isolated from cells using TRIzol reagent (Thermo Fisher Scientific) according to the manufacturer's protocol. cDNA was generated from RNA samples using Prime Script RT Master Mix. Real-time quantitative PCR (qPCR) was carried out using mixtures of SYBR Green PCR Master Mix (Thermo Fisher Scientific), cDNA, and forward and reverse primers on a qTOWER Real-time PCR Thermal Cycler (Analytik Jena, Jena, Germany). The PCR cycling parameters were as follows: 95 °C for 3 min, followed by 40 cycles of 95 °C for 10 s, 60 °C for 20 s, and 72 °C for 20 s and a final extension step of 20 s at 72 °C.

### Cellular uptake of SPMI-HIF2-1

The FITC fluorescent group was attached to the N-termini of peptides via aminohexanoic acid. HCT116 cells were seeded in laser confocal-specific dishes (35 mm, 6 × 10^4^ cells/dish) and incubated overnight. Cells were then treated with 10 µM FITC-PMI-HIF1-1, FITC-SPMI2, or FITC-SPMI-HIF2-1 without FBS for 6 h respectively. After washing the cells with PBS, cells were fixed with 4% (w/v) paraformaldehyde for 10 min and the nuclei were stained with DAPI. Imaging was performed using an LSM 510 Zeiss Axiovert 200M (v4.0) confocal microscope. The images were analyzed using LSM Image Viewer.

### Cell cycle analysis

HCT116 cells were seeded in 24-well plates (1 × 10^5^ cells/well) and cultured overnight in serum-deprived medium to synchronize the cell cycle. After the appropriate treatment, cells were harvested and washed with cold PBS. The cell cycle distribution was detected with a cell cycle staining kit (MultiSciences, Hangzhou, China) according to the manufacturer's instructions. Samples were detected by flow cytometry (Cytoflex, Beckman, USA).

### Apoptosis analysis

Apoptosis was examined with an Annexin V-FITC Apoptosis Detection Kit (BD Biosciences, USA). HCT116 cells were treated for 24 h with peptides at the indicated concentrations before staining with Annexin V-FITC and propidium iodide. After incubation at room temperature for 30 min in the dark, apoptosis was analyzed by flow cytometry (Cytoflex, Beckman, USA).

### Fluorescence polarization

An FP-based competitive or binding assay was established using ^25-109^MDM2, ^24-108^MDMX, and a fluorescently tagged p53-derived peptide as previously described. 34-36 For dose-dependent competitive binding experiments, MDM2 or MDMX protein (40 nM) was first incubated with TAMRA-p53 peptide (20 nM) in PBS (pH 7.4) on a Costar 96-well plate, to which a serially diluted solution of test peptide was added to a final volume of 125 μL. After 30 min of incubation at room temperature, the FP values were measured at λ_ex_ = 530 nm and λ_em_ = 580 nm on a Tecan Infinite M1000 plate reader. For binding experiments, FITC-labeled SPMI-HIF2-1 (20 nM) was incubated with different concentrations of MDM2, MDMX, and VHL proteins. Curve fitting was performed using GraphPad Prism software, and Ki values were calculated as described previously 37. Three replicates and three independent experiments were performed.

### α-Helicity

Peptides were dissolved in PB (pH = 7.2) to concentrations ranging from 10 to 50 µM. CD spectra were obtained on a Jasco J-715 spectropolarimeter at 20°C. The spectra were collected using a 0.1 cm path length quartz cuvette with the following measurement parameters: wavelength, 185-255 nm; step resolution, 0.1 nm; speed, 20 nm/min; accumulations, 6; bandwidth, 1 nm. The helical content of each peptide was calculated as reported previously 38.

### Proteolytic stability

Peptides were dissolved in PBS (50 mM, pH = 7.4) to a final concentration of 1 mM. α-Chymotrypsin was dissolved in PBS (50 mM, containing 2 mM of CaCl_2_, pH = 7.4) to a final concentration of 5 ng/μl. Then the peptide solutions (1 ml) were incubated with trypsin solution (10 μl) at 25°C. Samples were taken at 0, 5, 30, 60, 90, and 120 min and reactions were quenched with 20 μL of hydrochloric acid (1 M). The solution of the trypsin peptide fragments was monitored by HPLC at different time points to determine the fraction of protease degradation.

### Treatment of tumor xenografts *in vivo*

Four-week-old male BALB/c nude mice (15-20 g) were used to establish subcutaneous and orthotopic xenograft models. A total of 5 × 10^6^ HCT-116 cells were subcutaneously inoculated into the right flanks of the mice. Tumor volume was calculated using the following formula: V = (L × W^2^)/2, where L is the length and W is the width of the tumor nodules measured with a vernier caliper. When the volume of the tumors reached 75-100 mm^3^, the mice were randomly divided into four groups: saline, PMI-HIF1-1 (50 mg/kg), SPMI2 (50 mg/kg), and SPMI-HIF2-1 (10 mg/kg). Drugs were intravenously injected into the mice every 2 days for 2 weeks. In addition, the model of orthotopic CRC xenografts was established. Briefly, mice were anesthetized after fasting for 12 h. Subsequently, the distal anal and rectal mucosa were exposed, and transanal injection of HCT-116 cells (5 × 10^6^) was performed using a 30 G sterile needle. The injection was given directly into the rectal mucosa. Tumor growth was investigated, and once the tumors reached ~0.5 cm in size, the mice were randomly divided into two groups. The mice were treated every other day for 2 weeks with tail vein injections of either vehicle (saline) or sPMI-HIF2-1 (2 mg/kg). Body weight was measured before saline or drug injection. All mice were euthanized on day 14 following drug treatment, and the tumors were isolated, photographed, weighed, and measured.

### Histology and immunohistochemistry

To further confirm the antitumor activities of SPMI-HIF2-1 *in vivo*, histological analysis was performed on the CRC xenografts. Briefly, the samples were fixed in a 10% formalin solution, processed, embedded, sectioned, and either HE- or TUNEL-stained to reveal tumor tissue necrosis or IHC-stained using antibodies against p53, p21, MDM2, or MDMX. The HE, TUNEL, and IHC images were obtained on a Leica DMI3000 B phase-contrast microscope.

### Pharmacokinetic study

According to the randomization principle, male ICR mice (18-22 g) were divided into three groups (*n* = 5 mice/group): PMI-HIF1-1, SPMI2, and SPMI-HIF2-1. The peptides (1.8 mg/kg) were individually dosed in mice intravenously via tail vein injection and the blood samples were collected from the orbital venous plexus at 0, 0.5, 1, 2, 4, 6, 10, 24, and 48 h. All blood samples were centrifuged at 3000 rpm for 10 min, and the obtained plasma samples were immediately frozen at -80 °C until use. The standard curve was established as follows: Stock solutions of PMI-HIF1-1 and SPMI-HIF2-1 dissolved in acetonitrile were spiked into control plasma at final concentrations of 4000, 2000, 1000, 500, 250, 125, 62.5, 31.25, 15.625, 7.8125, 3.90625, 1.953125, and 0.9765625 ng/ml. Stock solutions of SPMI2 dissolved in acetonitrile were spiked into control plasma at final concentrations of 8000, 4000, 2000, 1000, 500, 250, 125, 62.5, 31.25, 15.625, 7.8125, 3.90625, 1.953125, and 0.9765625 ng/ml. Plasma samples (30 μL) were treated with 90 μL acetonitrile for protein precipitation. Then all samples were vortexed for 2 min and centrifuged for 15 min at 13000 rpm and 4°C. The supernatants were analyzed by ultraperformance liquid chromatography-electrospray ionization-tandem triple quadrupole mass spectrometry (UPLC-ESI-QqQ-MS, Shimadzu 8040). The system was equipped with an XBridge BEH C18 column (2.5 μm, 2.1×100 mm; Waters, Milford, MA, USA). The column temperature was set at 35°C. The injection volume was 4 μl. Elution of the analytes was achieved with a gradient of 0.1% formic acid in water (buffer A) and 0.1% formic acid in acetonitrile (buffer B) as follows: 0-3 min 5%-95% B, 3-5 min 95% B, 5-5.1 min 95%-5% B, 5.1-7 min 5% B. For the preparation of standard curves, the areas under the curve of the respective MS peaks (PMI-HIF1-1 [1058/1001 *m*/*z*], SPMI2 [1445.85/1417.75 *m*/*z*], and SPMI-HIF2-1 [1125.4/1445.85 *m*/*z*]) were determined by integration of the peaks in the base peak intensity chromatograms and plotted against the concentration. The standard curves were as follows: PMI-HIF1-1 (*y* = 82.3292 *x* - 97.5172, R^2^ = 0.9980409), SPMI2 (*y* = 1.05703 *x* + 8.72856, R^2^ = 0.9934009), SPMI-HIF2-1 (*y* = 9.53684 *x* - 174.28, R^2^ = 0.9964491). The plasma concentration at each time point was calculated from the extracted peak area of the same mass peaks of each peptide using the corresponding standard curve.

### Structural modeling

The structures of MDM2 (PDB: 6Y4Q), MDMX (PDB: 3EQY), and VHL (PDB: 1LM8) were picked as the initial models. The nonstandard peptide structures were sketched using Discovery Studio Visualizer 2021. MDM2 and VHL were docked by LightDock 0.9.1 with default parameters. Peg was then attached to the nonstandard peptide structure to form ternary complexes. Then, a short molecular docking simulation was performed to minimize this complex by 500 ps at constant pressure and temperature with a time step of 1 fs. Nonstandard amino acid parameters were determined using tleap, which is a program in AmberTools. A cutoff of 10 Å was used for nonbonded interactions and long-range electrostatic interactions were treated with the Particle Mesh Ewald method. All figures were drawn using PyMOL.

## Results and Discussion

### Design and synthesis of SPMI-based SP-PROTACs

**Figure 1:**
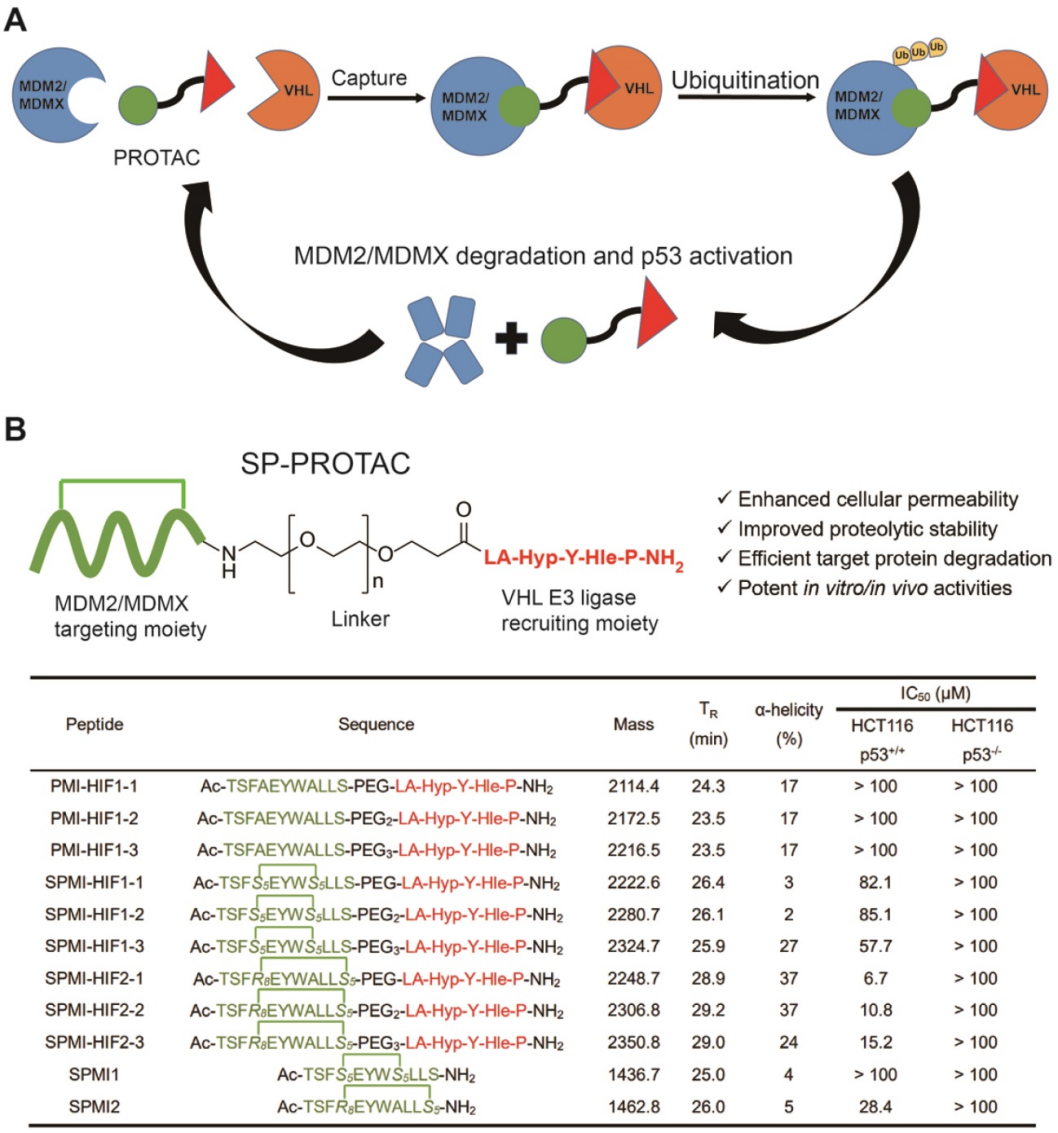
** Mechanism of PROTACs and the design of SP-PROTACs targeting MDM2 and MDMX. (A)** Mechanism through which PROTACs induce MDM2 and MDMX degradation. **(B)** Design and the sequences of SP-PROTACs targeting MDM2 and MDMX reported in this study. SP-PROTAC could simultaneously exhibit improved cellular permeability and proteolytic stability attributed to the stapled peptides, and efficient target protein degradation to the PROTACs. The designed peptide sequences, mass data, retention time (T_R_), α-helicity, and the IC_50_ values against HCT116 p53^+/+^ and HCT116 p53^-/-^ cell lines are shown. The green blocks indicate the MDM2/MDMX targeting motif and the red blocks indicate the VHL E3 ligase-recruiting motifs. Compared to the linear peptide and/or common stapled peptides, SP-PROTACs showcased advantages including enhanced cellular uptake, improved proteolytic stability, efficient target protein degradation, and potent *in vitro/in vivo* activities.

Conventional PROTAC molecules are made up of an MDM2/MDMX binding motif, an E3 ligase-recruiting ligand, and a suitable linker. Such PROTACs would be expected to capture MDM2/MDMX and VHL at the same time, thereby triggering the subsequent ubiquitination and proteasome-mediated degradation of target proteins **(Figure 1A)**. In our study, the rationally designed SP-PROTACs, consisting of MDM2/MDMX stapled peptide antagonists and VHL binding motifs, could simultaneously show the advantages of both stapled peptides and PROTAC bifunctional molecules. Lane and colleagues previously designed and synthesized the *i, i+7* stapled PMI-N8A peptide, which induced a specific p53 response but failed to drive the cell to undergo p53-dependent apoptosis 18. In that case, *R*-octenyl-alanine (R8)-*S*-pentenyl-alanine (S5) paired amino acids were installed at the *i, i+7* position for stapling two helix turns. For stapling a single helix turn, a combination of S5-S5 would be introduced at the *i, i+4* position. Accordingly, we designed the *i, i+4* or *i, i+7* stapled PMI-N8A peptide, termed SPMI1 and SPMI2, respectively, for binding with MDM2 and MDMX **(Figure 1B)**. To recruit VHL, the hydroxyproline (Hyp)- and homoleucine (Hle)-containing hexapeptide (LA-Hyp-Y-Hle-P), termed HIF, was chosen as previously described 28. The hydroxyl group of Hyp in the VHL ligand is critical for its binding with VHL and herein we thus used Hyp instead of the common Pro motif 31. Additionally, the hydrophobic cave of VHL with the common Ile residue occupied allowed for the introduction of another residue, such as Hle, with a longer side chain, as determined through structural modeling analysis. Overall, the introduction of Hyp and Hle not only improved the binding between HIF and VHL, but also enhanced the proteolytic resistance of HIF peptide. Considering the potential solubility problem, we used polyethylene glycol (Peg) of different lengths as the linker to bridge SPMI and HIF peptides, thus affording a series of SPMI-based SP-PROTACs (**Figure 1B**). The synthesis of SP-PROTACs was accomplished through the assembly of a linear peptide sequence by standard fluorenylmethyloxycarbonyl (Fmoc)-based solid phase peptide synthesis (SPPS), followed by ring closing metathesis (RCM) cyclization in presence of first-generation Grubbs' catalyst. The detailed synthetic route is shown in **Figure S1**. The crude peptides cleaved from the resin were further purified by reverse-phase high-performance liquid chromatography (RP-HPLC) and verified by HPLC (>95%) and high-resolution mass spectrometry (HR-MS) (**Table S1** and **Supplementary Materials**).

### Functional characterization of SP-PROTACs *in vitro*

**Figure 2:**
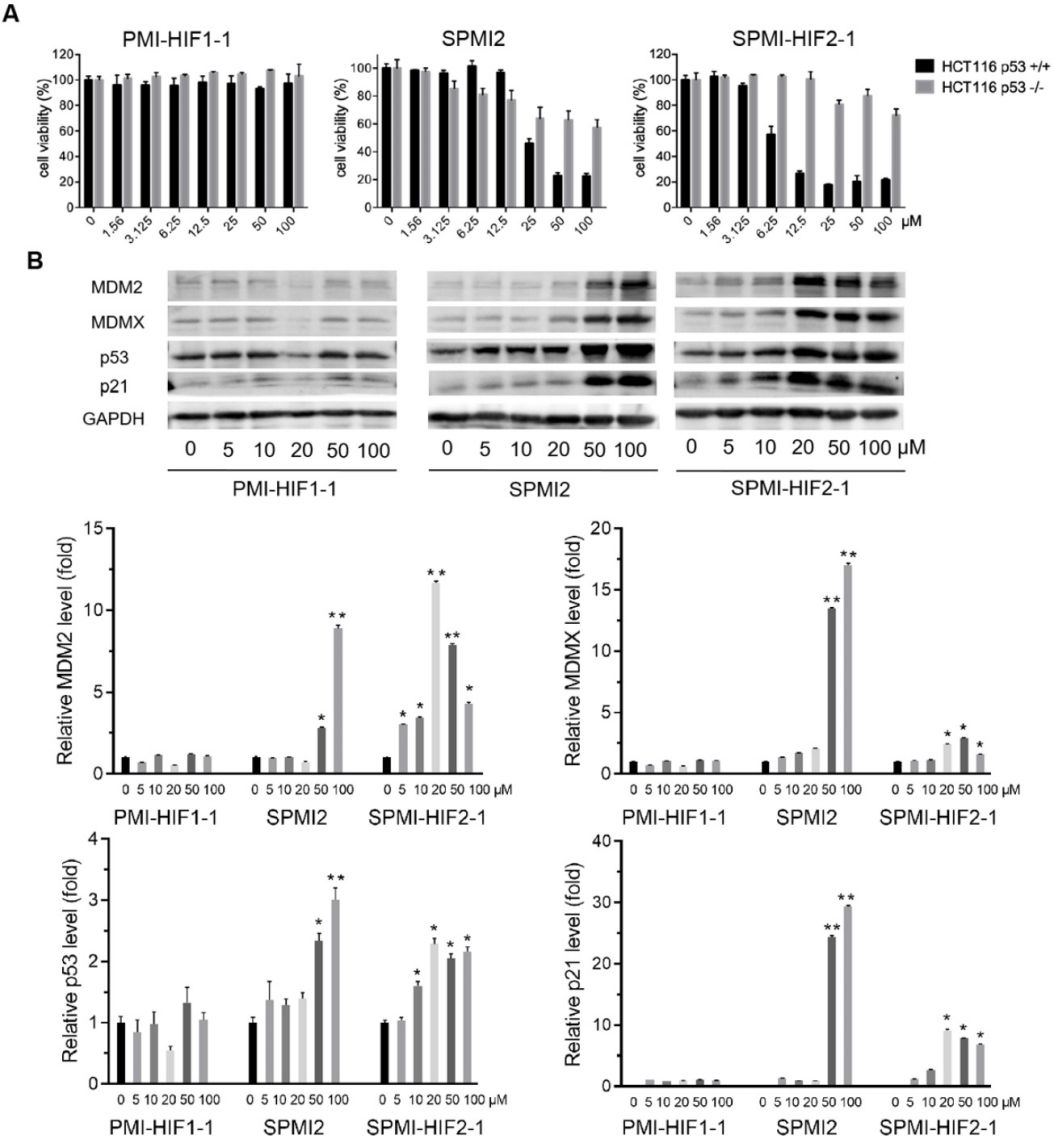
** SPMI-HIF2-1 effectively killed HCT116 p53^+/+^ but not p53^-/-^ cells via a simultaneous atypical degradation of both MDM2 and MDMX and durable p53 activation. (A)** Cell viability of representative PMI-HIF1-1, SPMI2, and SPMI-HIF2-1 against HCT116 p53^+/+^ and p53^-/-^ cancer cells. The experiment was performed using a Cell Counting Kit-8 (CCK-8). **(B)** The protein levels of MDM2, MDMX, p53, and p21 were analyzed by western blot analysis after HCT116 p53^+/+^ cells were treated with PMI-HIF1-1, SPMI2, and SPMI-HIF2-1 at various concentrations. Data are shown as the mean ± SEM of three independent experiments. *P*-values were calculated using the *t*-test (*P < 0.05; **P < 0.01).

Next, we evaluated the antitumor activities of the above SP-PROTACs. Human colon cancer HCT116 cells were exposed to our compounds and *TP53*-deficient HCT116 p53^-/-^ cancer cells were used as the negative control. The data are shown in **Figures 2A** and **S2**. Not surprisingly, the linear peptide-based PROTACs, including PMI-HIF1-1, PMI-HIF1-2, and PMI-HIF1-3, failed to kill both HCT116 p53^+/+^ and HCT116 p53^-/-^ cancer cells even at a concentration of 100 μM. Introduction of the HIF motif slightly improved the antitumor activities of SPMI1, in which SPMI-HIF1-1, SPMI-HIF1-2, and SPMI-HIF1-3 showcased moderate cytotoxicity against HCT116 p53^+/+^ cells with IC_50_ values of 82.1, 85.1, and 57.7 μM, respectively, suggesting that *i, i+4* stapling is not a good choice for PMI derivation. Another *i, i+7* stapled SPMI2 peptide displayed a better dose-dependent inhibitory effect against HCT116 p53^+/+^ cells than the SPMI1 peptide with an IC_50_ value of 28.4 μM. Of note, when combined with the HIF motif bridged with different Peg linkers, the corresponding SP-PROTACs showcased obviously enhanced inhibitory activities against HCT116 p53^+/+^ cells. IC_50_ values of SPMI-HIF2-1, SPMI-HIF2-2, and SPMI-HIF2-3 were 6.7, 10.8, and 15.2 μM, respectively, among which the best SP-PROTAC, SPMI-HIF2-1, acquired a nearly 5-fold and over 20-fold enhancement compared with SPMI2 and PMI-HIF1-1, respectively (**Figures 1B and 2A**). Additionally, no obvious cytotoxicity against HCT116 p53^-/-^ cells was observed for SPMI-HIF2-1 even at a concentration of 100 μM, indicating excellent selectivity and potential p53 pathway reactivation. Theoretically the chiral center of the Hyp residue in the VHL binding moiety is critical for its binding with E3 ligase. We thus used the (*S*)-configuration diastereoisomer of SPMI-HIF2-1, termed SPMI-HIF2-1S, as a negative control, and evaluated its antiproliferative activity. As shown in **Figure S2**, SPMI-HIF2-1S could kill HCT116 p53^+/+^ cells with an IC_50_ value of 13.4 μM, 2-fold higher than that of SPMI-HIF2-1 (6.7 μM).

Western blot analysis was conducted to confirm the functional mechanism of the optimized SPMI-HIF2-1. As expected, the linear peptide-based PROTAC PMI-HIF1-1 did not cause obvious changes in the protein levels of MDM2, MDMX, p53, and p21 in HCT116 p53^+/+^ cells. Dose-dependent induction of MDM2, MDMX, p53, and p21 became evident for SPMI2 at concentrations of 50 and 100 μM, which was consistent with the results of our cell viability assay (**Figure 2B**). For SPMI-HIF2-1, the protein levels of p53 and p21 were increased in a dose-dependent manner at concentrations ranging from 5 to 20 μM and reached a plateau at higher concentrations. Interestingly, SPMI-HIF2-1 could efficiently increase and then decrease the protein levels of both MDM2 and MDMX, indicative of an atypical degradation of both MDM2 and MDMX different from the conventional small molecule-based PROTAC. Against HCT116 p53^-/-^ cells, SPMI-HIF2-1 also showcased the atypical degradation of both MDM2 and MDMX (**Figure S3**), whereas SPMI2 induced slightly increasing protein levels of MDM2 and MDMX. A slightly increasing protein level of p21 could be observed upon treatment with both SPMI2 and SPMI-HIF2-1.

### SPMI-HIF2-1 induced the atypical degradation of both MDM2 and MDMX

**Figure 3:**
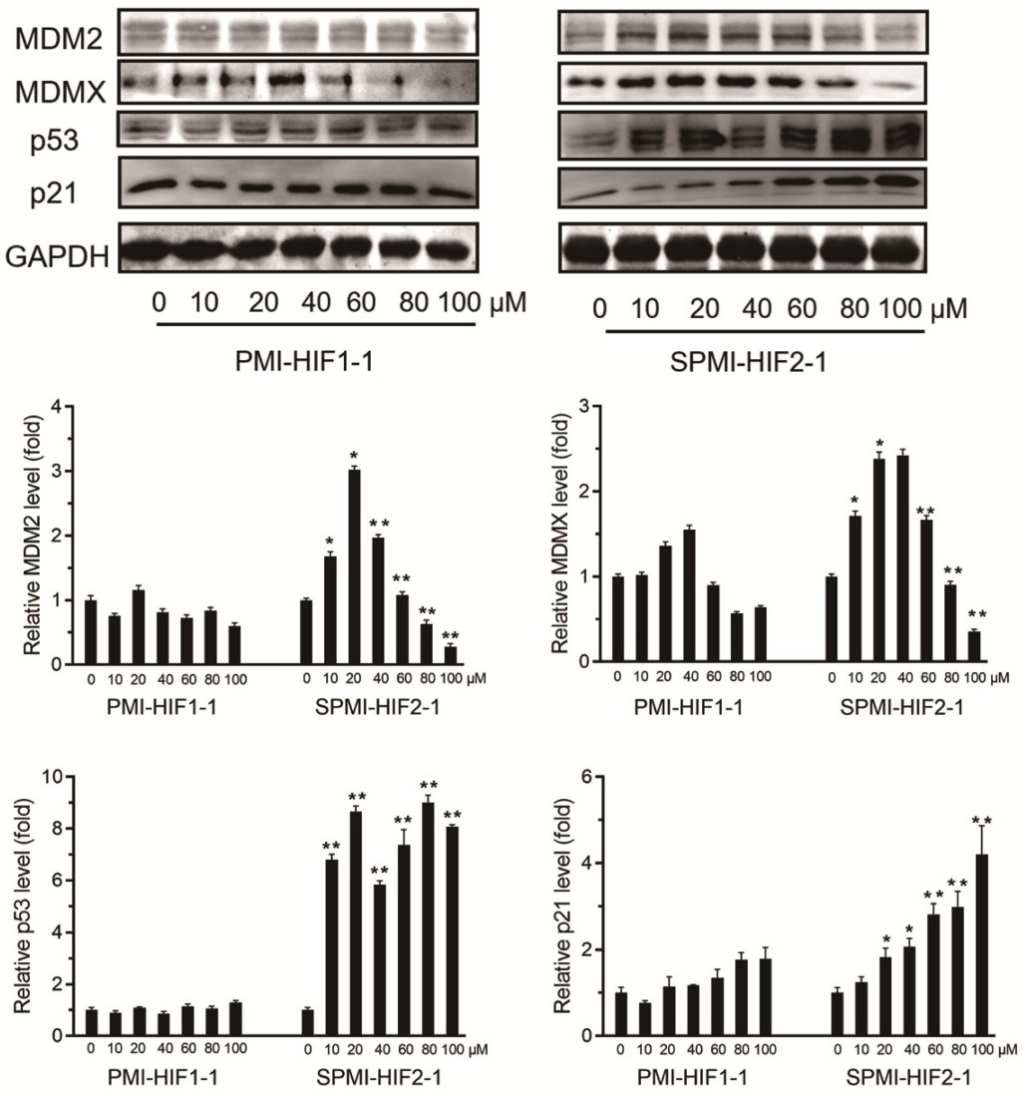
** SPMI-HIF2-1 induced atypical degradation of both MDM2 and MDMX.** SPMI-HIF2-1 increased the protein levels of both MDM2 and MDMX at concentrations below 20 µM and promoted target protein degradation in a dose-dependent manner at concentrations ranging from 20 to 100 µM. MCF-7 cells were treated with PMI-HIF1-1 and SPMI-HIF2-1 at various concentrations and the protein levels of MDM2, MDMX, p53, and p21 were analyzed by western blot analysis. Data are shown as the mean ± SEM of three independent experiments. *P*-values were calculated using the t-test (*P < 0.05; **P < 0.01).

To validate this atypical degradation triggered by our designed SP-PROTAC, we next tested the relevant protein levels of both MDM2 and MDMX in another cancer cell line, MCF-7. First, 10, 20, 40, 60, 80, or 100 μM SPMI-HIF2-1 was added to the culture medium of MCF-7 cancer cells, followed by incubation for 48 h and detection of the protein levels of MDM2 and MDMX. Similar to HCT116 cells, treatment with 20 μM SPMI-HIF2-1 induced the highest level of target protein in MCF-7 cells, and higher SPMI-HIF2-1 levels (20-100 μM) resulted in degradation of both MDM2 and MDMX in a dose-dependent manner (**Figure 3**). For both HCT116 and MCF-7 cells, the turning point of atypical degradation of MDM2 and MDMX was approximately 20 μM. Additionally, the SPMI-HIF2-1-induced degradation of MDM2 was more efficient than that of MDMX in both cell lines. SPMI-HIF2-1 could induce robust activation of p53 in MCF-7 cells, whereas PMI-HIF1-1 did not affect p53 protein levels (**Figure 3**). Additionally, a dose-dependent elevation of p21 protein levels could be observed in the SPMI-HIF2-1 group, but no obvious change was detected in the linear peptide group (**Figure 3**).

To confirm that the degradation of MDM2 and MDMX by SPMI-HIF2-1 was proteasome-dependent, we next pretreated the cells with the proteasome inhibitor epoxomicin (EPI) 39. As shown in Figure S4, the degradation of both MDM2 and MDMX in HCT116 and MCF-7 cells by SPMI-HIF2-1 at concentrations of 50 and 100 μM could be blocked by EPI. We also evaluated the mRNA levels of *MDM2* and *MDMX* in HCT116 and MCF-7 cells after treatment with SPMI-HIF2-1. The mRNA levels of *MDM2*/*MDMX* in both cell lines increased in a dose-dependent manner or not obviously (**Figure S5**), indicating that our designed SP-PROTAC indeed affected the target protein levels, rather than the mRNA levels.

Next, we investigated the protein levels of MDM2 and MDMX in other cancer cell lines with different p53 genetic status, including A549, U87, HCT116 p53^-/-^, and U251 cells. As shown in **Figures S3** and **S6**, initially increasing and then gradually decreasing protein levels of MDM2 and MDMX were observed. Overall, our optimized SP-PROTAC resulted in a higher degradation efficiency of MDM2 than MDMX, and the MDM2/MDMX degradation efficiency was higher in p53 wild-type cancer cells than in p53-mutated or -deficient cells. Taken together, the above results indicate that the optimized SP-PROTAC SPMI-HIF2-1 inhibits the growth of cancer cells by promoting the atypical degradation of both MDM2 and MDMX in a proteasome-dependent manner and reactivating the p53 pathway.

### SPMI-HIF2-1 penetrated cell membranes and induced cell cycle arrest and apoptosis

**Figure 4:**
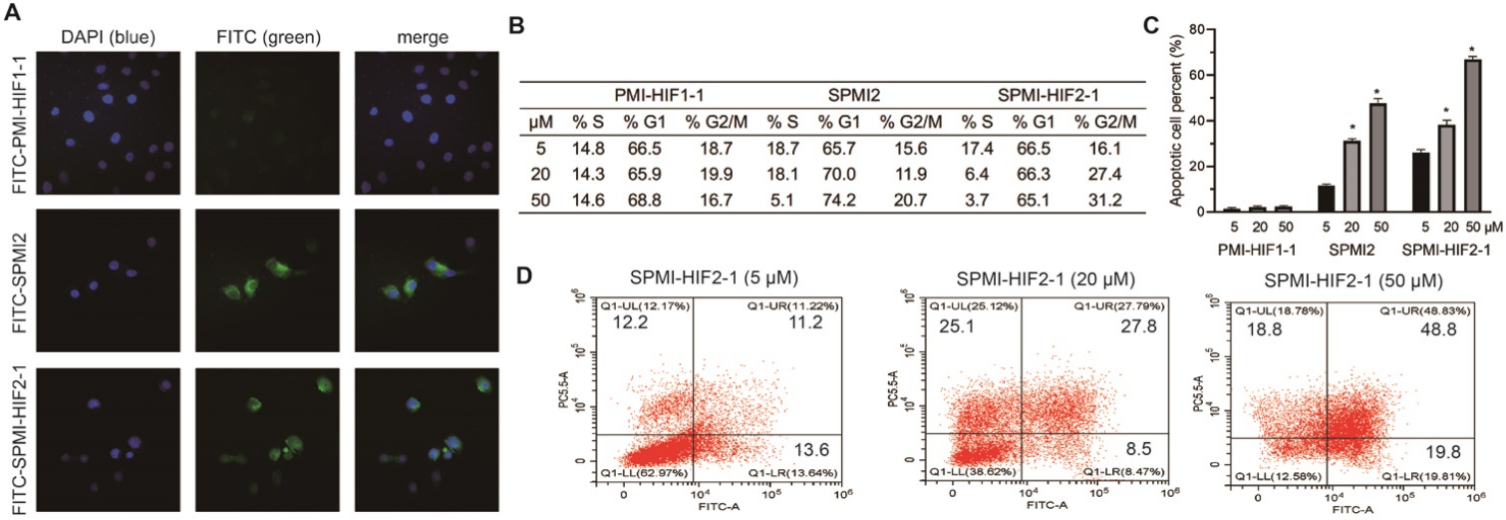
** SPMI-HIF2-1 could efficiently penetrate cell membranes and induce cell cycle arrest and apoptosis. (A)** Representative images of FITC-labeled PMI-HIF1-1, SPMI2, and SPMI-HIF2-1 (10 µM) localization in HCT116 cells as determined by confocal microscopy. The FITC fluorescent group was attached to the N-terminus of peptides via aminohexanoic acid. Imaging was performed using an LSM 510 Zeiss Axiovert 200M (v4.0) confocal microscope and images were analyzed using LSM Image Viewer. Peptide stapling could effectively aid the peptides to penetrate cell membranes. **(B)** SPMI-HIF2-1 caused cell cycle arrest in HCT116 cancer cells. The cell cycle distribution was detected with a cell cycle staining kit. **(C and D)** Apoptotic response to PMI-HIF1-1, SPMI2, and SPMI-HIF2-1 in HCT116 cells as analyzed by flow cytometry. HCT116 cells were exposed to PMI-HIF1-1, SPMI2, and SPMI-HIF2-1 (5, 20, or 50 µM) for 48 h. Apoptosis was examined with an Annexin V-FITC Apoptosis Detection Kit. The cell cycle distribution and apoptosis were detected by flow cytometry. Data are shown as the mean ± SEM of three independent experiments. *P*-values were calculated using the t-test (*P < 0.05).

Peptide stapling has been proven to be capable of enhancing the cellular uptake of peptides 15. Therefore, we evaluated the ability of SPMI-HIF2-1 to cross membranes by confocal microscopy. PMI-HIF1-1 and SPMI2 were used as the controls. PMI-HIF1-1, SPMI2, and SPMI-HIF2-1 were N-terminally conjugated to the fluorescein isothiocyanate (FITC) motif, followed by the addition to HCT116 cells. As shown in **Figure 4A**, a diffuse intracellular localization was observed for SPMI2 and SPMI-HIF2-1, but not PMI-HIF1-1, confirming the ability of stapled peptides to cross cellular membranes.

Next, we evaluated cell cycle arrest and apoptosis, which are considered as the most important cellular functions of p53. As shown in **Figures 4B** and **S7**, SPMI-HIF2-1 effectively caused cell cycle arrest in the G2/M phase in a dose-dependent manner, leading to the gradual depletion of cells in the S phase. Such phenomenon could be observed for SPMI2 at high concentrations but not for the linear PMI-HIF1-1. Next, the Annexin V assay was performed to quantify the apoptotic effects of SPMI-HIF2-1 (**Figures 4C** and **D** and **S8)**. Dose-dependent induction of apoptosis could be observed for both SPMI2 and SPMI-HIF2-1, with a higher potency for SPMI-HIF2-1.

### Binding affinity, helicity, and proteolytic stability of SPMI-HIF2-1

**Figure 5:**
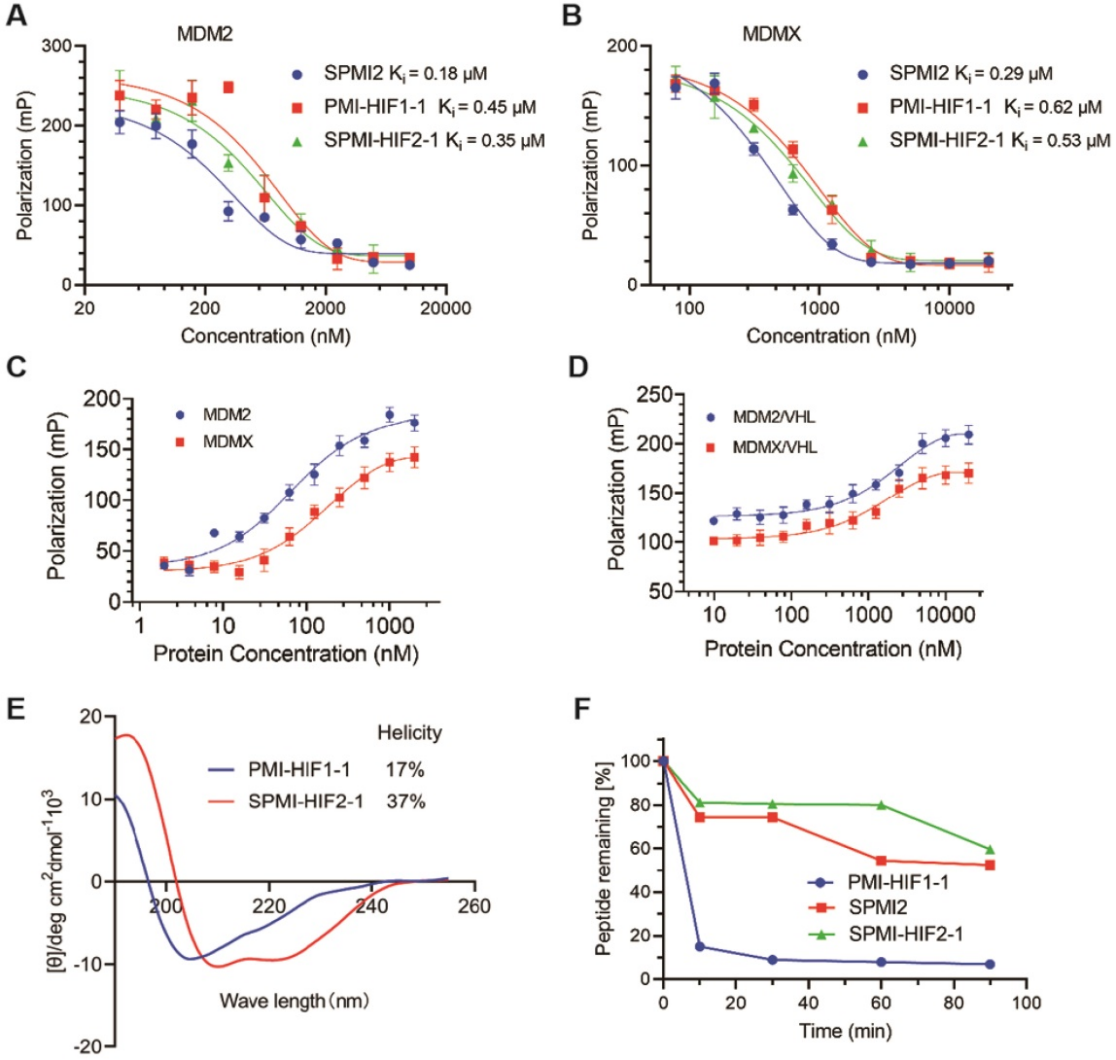
** Binding, helicity, and proteolytic stability of the optimized SP-PROTAC. (A and B)** Quantification assays of the interactions of MDM2 and MDMX with PMI-HIF1-1, SPMI2, and SPMI-HIF2-1 by FP-based competitive binding assays. MDM2 or MDMX protein (40 nM) was first incubated with FITC-labeled p53 peptide (20 nM) in PBS (pH 7.4), to which a serially diluted solution of test peptide was added to a final volume of 125 µL. **(C)** The interactions of MDM2 and MDMX with SPMI-HIF2-1 as determined by FP-based binding assays. Different concentrations of MDM2 or MDMX protein were incubated with FITC-labeled SPMI-HIF2-1 (20 nM) in PBS (pH 7.4). (D) MDM2 or MDMX protein (200 nM) was first incubated with FITC-labeled SPMI-HIF2-1 (20 nM), to which a serially diluted solution of VHL protein was added to a final volume of 125 µl. Three replicates and three independent experiments were performed; each curve is the mean of three independent measurements. FP values were measured at λ_ex_ = 530 nm and λ_em_ = 580 nm on a Tecan Infinite M1000 plate reader. Curves were fitted and Ki values were calculated using GraphPad Prism software. **(E)** Representative CD spectra of PMI-HIF1-1 and SPMI-HIF2-1. The circular dichroism (CD) spectra were obtained on a Jasco J-715 spectropolarimeter at 20°C. The helicities of these peptides were calculated based on the values of [θ]_222_. **(F)** Proteolytic stability of PMI-HIF1-1, SPMI2, and SPMI-HIF2-1 against chymotrypsin. The percentages of the remaining intact peptides were detected by HPLC at 0, 5, 30, 60, 90, and 120 min.

After verification of the cellular uptake of SP-PROTAC, we investigated its binding affinity, helicity, and proteolytic stability. We first tested the binding affinity of the optimized SP-PROTAC with MDM2 and MDMX using an FP-based competitive binding assay. As shown in **Figure 5A**, SPMI-HIF2-1 bound with MDM2 with a similar Ki value (0.35 μM) as PMI-HIF1-1 (0.45 μM). It is not strange that SPMI-HIF2-1 displayed a lower binding potency than its stapled counterpart SPMI2 (Ki = 0.18 μM), due in part to the introduction of the HIF motif. Similar trends were observed for MDMX in spite of a weaker binding potency than MDM2; the binding Ki values of PMI-HIF1-1, SPMI2, and SPMI-HIF2-1 were 0.29, 0.62, and 0.53 μM, respectively (**Figure 5B**). The above findings showed that the PROTAC molecule could basically maintain the binding potency of peptide with the target protein, and SP-PROTAC exhibited a stronger binding affinity with MDM2 and MDMX than the linear peptide-based PROTAC.

To investigate the formation of ternary complexes, we next performed binding assays of FITC-labeled SPMI-HIF2-1 with the target protein and E3 ligase VHL. As shown in **Figure 5C**, FITC-labeled SPMI-HIF2-1 could bind with MDM2 or MDMX with a similar binding potency. Then we incubated FITC-labeled SPMI-HIF2-1 (20 nM) with MDM2 or MDMX protein (200 nM), followed by the addition of different concentrations of VHL protein. As shown in **Figure 5D**, a dose-dependent elevation of polarization values could be observed for both MDM2 and MDMX, indicative of the potential cooperativity between the E3 ligase and target protein in forming the E3-PROTAC-target protein ternary complex.

Generally, the helical contents of peptides are positively correlated with their binding potency with target proteins. Circular dichroism (CD) spectroscopy was performed to determine the helical contents of SP-PROTACs. The corresponding helicities are tabulated in **Figure 1B**. Helical peptides exhibited characteristic peaks in the CD spectra, with minimum values at 208 and 222 nm. Not surprisingly, all *i, i+7* SP-PROTACs displayed higher helical contents than their linear counterparts (**Figures 5E** and** S9**). Among them, SPMI-HIF2-1 exhibited a 37% helicity, whereas the helicity of PMI-HIF1-1 was only 17%. These results could partly explain the binding affinity of these two PROTAC-based peptides.

To evaluate the effect of hydrocarbon peptide stapling on the proteolytic stability of SP-PROTACs, the optimized SPMI-HIF2-1 was selected for enzymatic digestion in the presence of chymotrypsin protease, and the residual contents were detected by HPLC at different time points. As shown in **Figure 5F**, the degradation of SPMI-HIF2-1 was significantly slower than that of PMI-HIF1-1, similar to SPMI2. More than 60% of SPMI-HIF2-1 could still be observed after proteolytic digestion for 90 min, whereas more than 80% of PMI-HIF1-1 was digested within 10 min. The above results indicate that SP-PROTACs could show better binding affinity, higher helical contents, and improved proteolytic stability than their linear peptide-based PROTAC counterparts.

### Efficient tumor inhibition by SPMI-HIF2-1 in subcutaneous and orthotopical CRC xenograft models

**Figure 6:**
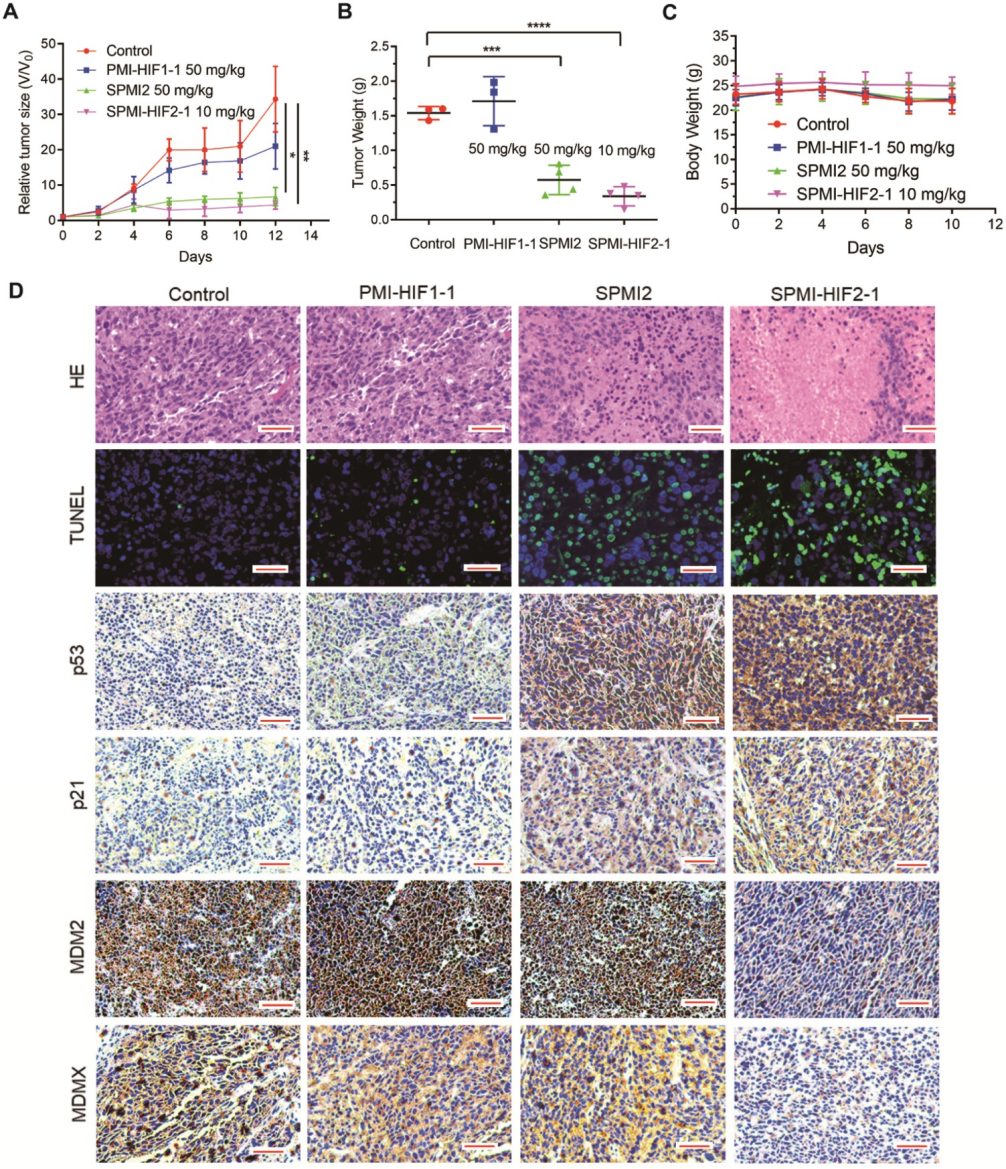
** SPMI-HIF2-1 inhibited tumor growth in a subcutaneous xenograft model.** HCT116 cells were inoculated into the right flanks of mice to establish the subcutaneous xenograft model. Mice were then randomly divided into four groups and treated with saline solution, PMI-HIF1-1 (50 mg/kg), SPMI2 (50 mg/kg), or SPMI-HIF2-1 (10 mg/kg) via tail vein injection every 2 days for 2 weeks. **(A and B)** Tumor size and weight were measured after the mice were sacrificed. *P*-values were calculated using the *t*-test (*P < 0.05; **P < 0.01). **(C)** Body weights were recorded at the indicated days. Data are shown as the mean ± SD per group. **(D)** Representative HE, TUNEL, and IHC images for p53, p21, MDM2, and MDMX in tumor sections (scale bar: 50 µm). Significant apoptotic activity, increased p53 and p21 protein levels, and decreased MDM2 and MDMX protein levels were detected in the SPMI-HIF2-1 group. These results indicate that tumor inhibition was realized through the degradation of MDM2 and MDMX and subsequent p53 activation.

Based on the above results, SPMI-HIF2-1 was selected for further evaluation. We used two colorectal tumor models based on different sites of inoculation, including subcutaneous and intrarectal, to examine the therapeutic efficacy of SPMI-HIF2-1. In the first tumor model, primary tumors were constructed in BALB/c nude mice within 2 weeks, followed by a 2-week administration of control, PMI-HIF1-1 at 50 mg/kg, SPMI2 at 50 mg/kg, or SPMI-HIF2-1 at 10 mg/kg via the tail vein every other day. Tumor volume and body weight were measured at the time of administration. As shown in **Figure 6A**, the most pronounced inhibition of tumor growth was observed in the 10 mg/kg SPMI-HIF2-1 group, which was similar to that in the 50 mg/kg SPMI2 group, 5-fold enhanced compared to that in the control group, and 3-fold enhanced compared to that in the 50 mg/kg PMI-HIF1-1 group. The tumor weight results at the end of the experiment were in agreement with the tumor volume results. Treatment with 10 mg/kg SPMI-HIF2-1 resulted in the best inhibition of tumor growth, similar to 50 mg/kg SPMI-HIF2-1 (**Figure 6B**). Additionally, no obvious changes in body weight were observed in the control and treatment groups (**Figure 6C**), indicating a favorable safety profile of SPMI-HIF2-1.

The above results were confirmed by hematoxylin-eosin (HE) staining; the tumor area was reduced upon treatment with SPMI-HIF2-1 or SPMI2, in contrast to control or PMI-HIF1-1 treatment (**Figure 6D**). Moreover, TdT-mediated dUTP nick end labeling (TUNEL) immunofluorescence analysis showed that treatment with SPMI-HIF2-1 significantly increased the level of apoptosis compared to the PMI-HIF1-1 and control groups. Immunohistochemistry (IHC) assays revealed a sharp restoration of p53 and p21 levels in the SPMI-HIF2-1 and SPMI2 groups. Of note, a significant decrease in the protein levels of both MDM2 and MDMX in the SPMI-HIF2-1 group was detected, as shown in **Figure 6D**, suggesting that our SP-PROTAC exerted therapeutic effects *in vivo* through stimulating degradation of both MDM2 and MDMX and reactivating the p53 pathway.

**Figure 7:**
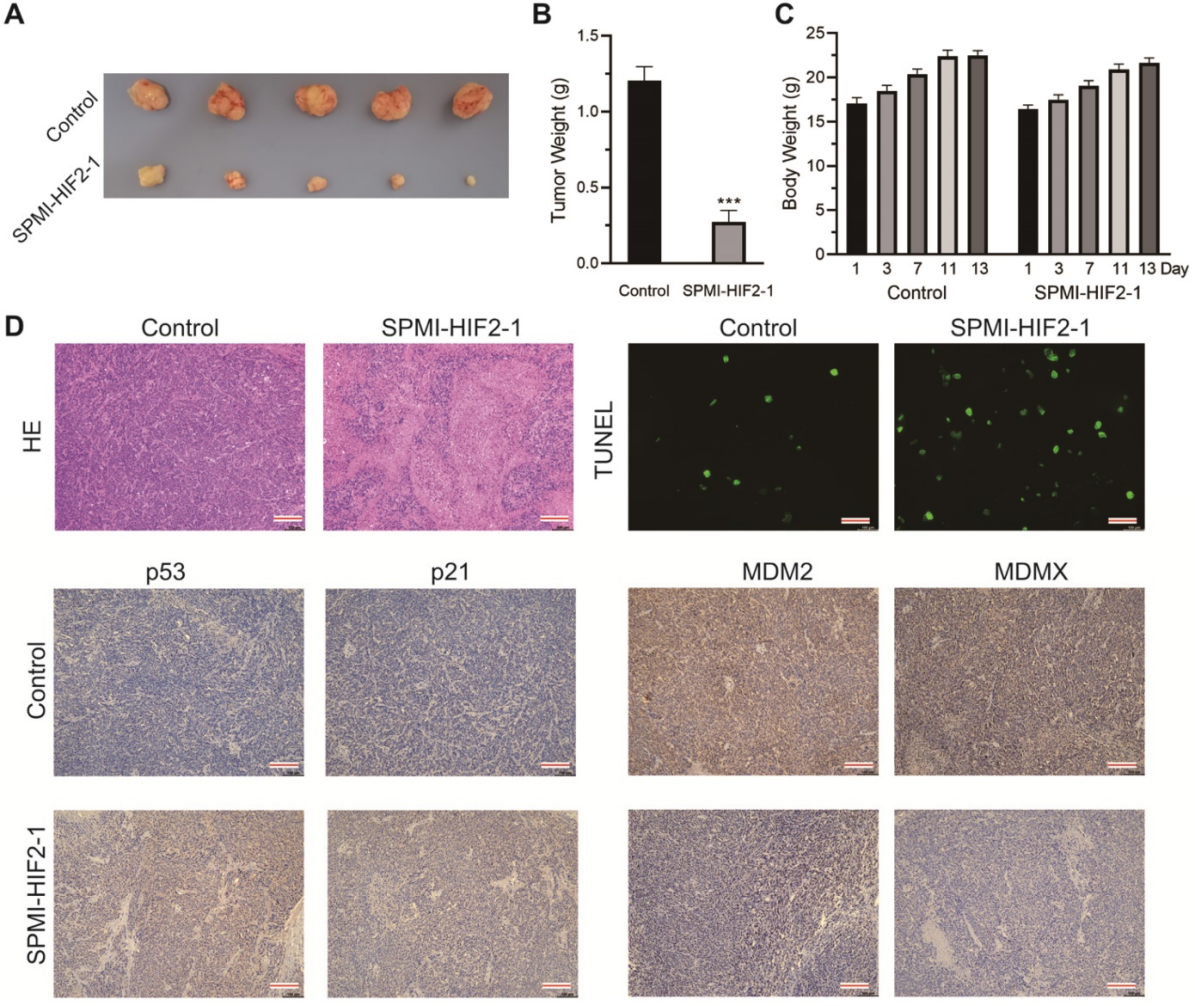
** SPMI-HIF2-1 inhibited tumor progression in a nude mouse CRC xenograft model.** CRC xenograft models were established through orthotopical implantation of HCT116 cells into the rectal mucosa of mice. Mice were then randomly divided into two groups (*n* = 5 mice/group) and treated with PBS or SPMI-HIF2-1 (2 mg/kg) via tail vein injection every 2 days until the 13th day. **(A)** Representative images of the tumors excised at the end of the experiment. **(B)** Tumor weight was measured after the mice were sacrificed. *P*-values were calculated using the *t*-test (***P < 0.001). **(C)** Body weights of the mice were recorded at the indicated days. Data for tumor weight and body weight are shown as the mean ± SD. **(D)** Representative HE, TUNEL, and IHC images for p53, p21, MDM2, and MDMX in tumor sections. Significant apoptotic activity, increased p53 and p21 protein levels, and decreased MDM2 and MDMX protein levels were detected in the SPMI-HIF2-1 group. These results indicated that tumor inhibition was realized through the degradation of MDM2 and MDMX and subsequent p53 activation (scale bar: 150 µm).

In the second xenograft model, two groups of intrarectal tumor-bearing mice (**Figure S10**) were treated with saline solution or SPMI-HIF2-1 via tail vein injection every 2 days until the 13th day. The body weights of the mice were monitored at the indicated days. SPMI-HIF2-1 treatment at a dose of 2 mg/kg significantly inhibited tumor growth, resulting in a 77% reduction in tumor weight compared to the control group (**Figure 7A and B**). Compared to the saline-treated controls, no obvious change in body weight was observed in the SPMI-HIF2-1 group (**Figure 7C**). At the end of the treatment, the mice were sacrificed, and the tumors were evaluated histologically by HE and TUNEL staining and IHC analysis (**Figure 7D**). HE and TUNEL staining revealed significant tissue death in the SPMI-HIF2-1 group compared to the saline control. IHC analysis revealed that the protein levels of p53 and p21 were upregulated in the SPMI-HIF2-1 group, whereas the protein levels of MDM2 and MDMX were downregulated. These above results strongly suggest that SPMI-HIF2-1 may be a potent therapeutic agent in different CRC xenograft models and SP-PROTACs could represent a new class of anticancer agents.

### Pharmacokinetic evaluation of SPMI-HIF2-1

The optimized SP-PROTAC and two parent peptides were then tested *in vivo* to assess the influence of peptide stapling and HIF introduction on their pharmacokinetic profiles. PMI-HIF1-1, SPMI2, or SPMI-HIF2-1 was injected intravenously via the tail vein in a single dose (1.8 mg/kg) into five mice, followed by the collection of blood samples over a period of 48 h.

**Figure 8:**
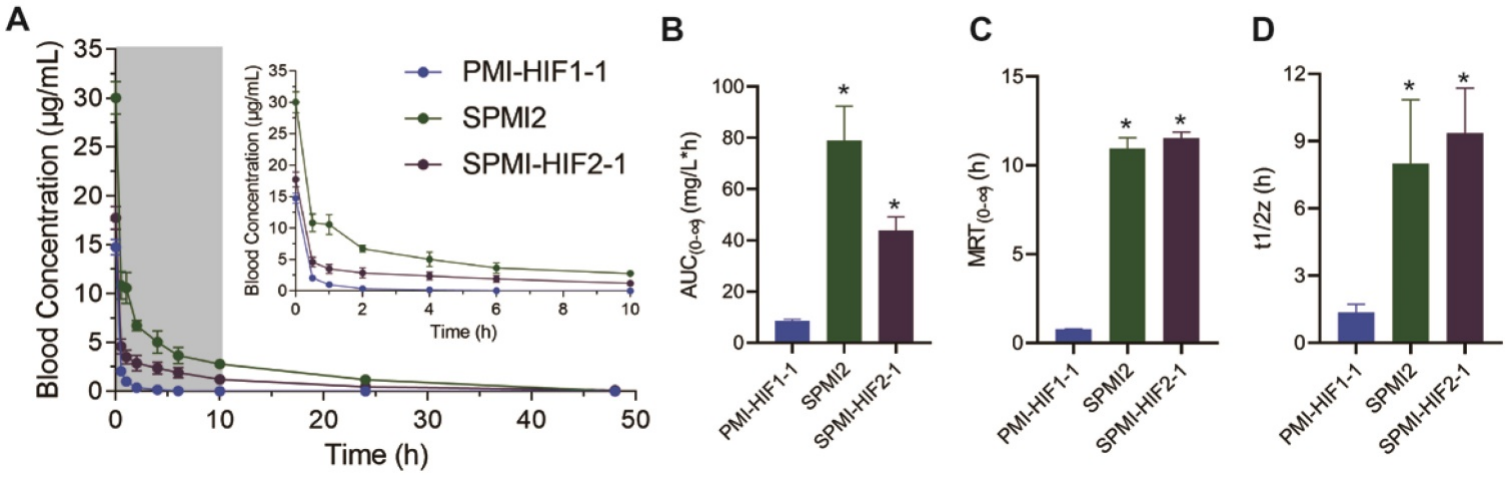
** Pharmacokinetic studies of PMI-HIF1-1, SPMI2, and SPMI-HIF2-1. (A)** Blood concentrations of PMI-HIF1-1, SPMI2, and SPMI-HIF2-1 at 0.5, 1, 2, 4, 6, 10, 24, and 48 h. **(B-D)** AUC, MRT, and half-time values of PMI-HIF1-1, SPMI2, and SPMI-HIF2-1. Male Institute of Cancer Research (ICR) mice (18-22 g) were divided into three groups (*n* = 5 mice/group): PMI-HIF1-1, SPMI2, and SPMI-HIF2-1. The peptides (1.8 mg/kg) were individually dosed in mice intravenously via tail vein injection. Blood samples were collected from the orbital venous plexus at 0, 0.5, 1, 2, 4, 6, 10, 24, and 48 h.

A single injection of SPMI-HIF2-1 resulted in an immediate spike in concentration (C_max_ = 17.7 μg/ml, mean value calculated) after injection (**Figure 8A**), following distribution and elimination phases, resulting in an AUC_(0-∞)_ value of 43.9 mg/L×h, an MRT of 11.5 h, and a half-life (t_1/2_) of 9.3 h (**Figure 8B-D**). By contrast, the linear PMI-HIF1-1 showed a C_max_ of 14.7 μg/ml in the plasma after administration, but an AUC_(0-∞)_ of 8.6 mg/L×h, an MRT of 0.7 h, and a terminal half-life (t_1/2_) of 1.3 h, which were dramatically lower than those of SPMI-HIF2-1. Nearly no remaining PMI-HIF1-1 peptide could be detected at 0.5 h after administration. Of note, another stapled counterpart, SPMI2, showcased a similar or even better pharmacokinetic profile than SP-PROTAC, with a C_max_ of 30.0 μg/ml in plasma after administration, an AUC_(0-∞)_ of 78.6 mg/L×h, an MRT of 10.9 h, and a half-life (t_1/2_) of 7.9 h. Considering these results for SPMI2 and SPMI-HIF2-1, it still remains challenging to prolong the circulation time and bioavailability of SP-PROTACs.

### Structural modeling of SPMI-HIF2-1 complexed with MDM2/MDMX and VHL

**Figure 9:**
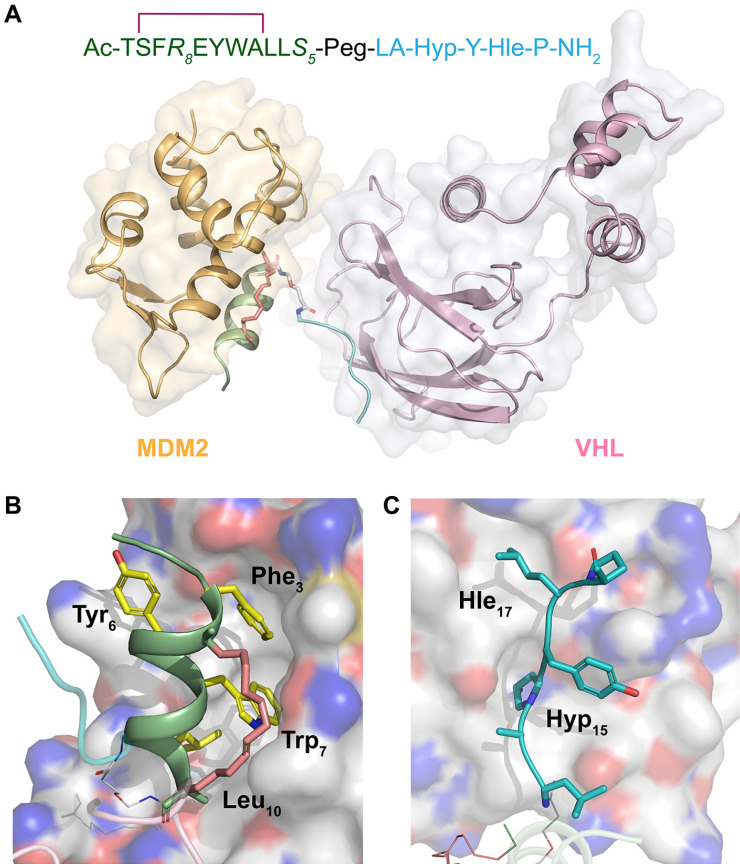
** Structural modeling analysis of the ternary complex of SPMI-HIF2-1, MDM2, and VHL. (A)** Overview of SPMI-HIF2-1 complexed with the target protein MDM2 and the E3 ligase VHL. The MDM2 binding motif is shown as a palegreen cartoon; a hydrocarbon staple is shown as salmon sticks; the Peg linker is shown as white sticks; and the VHL binding motif is shown as a cyan coil. **(B)** Binding surface between SPMI-HIF2-1 and MDM2. Yellow Phe3, Trp7, and Leu10 could be inserted into the MDM2 cave similarly to the PMI peptide.** (C)** Binding surface between SPMI-HIF2-1 and VHL. The key residues Hyp15 and Hle17 could be inserted into the VHL cave. The structures of MDM2 (PDB: 6Y4Q) and VHL (PDB: 1LM8) were picked as the initial models. A short molecular docking simulation was performed to minimize this complex.

To analyze the binding mode of SP-PROTAC with the target protein as well as E3 ligase, we performed docking studies of the ternary complexes consisting of SPMI-HIF2-1, MDM2/MDMX, and VHL. As shown in **Figure 9A**, SPMI-HIF2-1 bridged the gap and decreased the distance between MDM2 and VHL. Notably, in the ternary complex MDM2 and VHL did not interfere with each other, suggesting that in our case a simple Peg unit has a suitable linker length. This result was consistent with the *in vitro* and *in vivo* therapeutic efficiency. The binding modes of SPMI-HIF2-1 with MDM2 and VHL are shown in **Figure 9B** and **C**, respectively. In spite of the introduction of a stapling group, the SPMI2 motif in the SP-PROTAC bound tightly with MDM2 similarly to the parent PMI peptide, characterized by the hydrophobic interactions contributed by three key residues (Phe3, Trp7, and Leu10). Another HIF motif in SPMI-HIF2-1, adopting a random coil, could also be inserted into the cave of VHL. Two nonnatural amino acids (Hyp15 and Hle17) used in this study not only enhanced the relevant polar and nonpolar interactions, respectively, but also improved proteolytic resistance. Additionally, structural modeling analysis of the SPMI-HIF2-1-MDMX-VHL complex was conducted and a structural comparison between MDM2/MDMX complexes was made. As shown in **Figure S11**, SPMI-HIF2-1 binds with MDMX similarly as MDM2 with a negligible difference, which was consistent with the similar binding affinity of SPMI-HIF2-1 to MDM2 and MDMX. Structural analysis of ternary complexes involving PROTACs, target proteins, and E3 ligase may aid the further optimization of SP-PROTAC molecules.

## Conclusion

The emergence of PROTAC technology triggered the development of various functional small molecules with high degradation efficiency towards target proteins 26, 40. Unlike small molecule-based PROTACs, peptide-based PROTAC modalities were generally considered as undruggable due to the poor cellular uptake and proteolytic stability of conventional peptides 41. By combining peptide stapling with PROTAC technology, we developed a class of SP-PROTAC molecules targeting both MDM2 and MDMX. The first advantage of SP-PROTACs is the high binding potency and specificity towards the target protein. In this case, traditional small molecule PROTACs fail to target both MDM2 and MDMX, whereas SP-PROTACs represent a class of promising dual antagonists characterized by their large interaction faces. Another advantage of SP-PROTACs is the more accessible rational design and synthesis. The potential peptide sequence could easily be grafted from the important PPIs benefiting from the rapid development of structural biology and further synthesized using mature SPPS methods with high yields. One potential limitation of SP-PROTACs is that the resultant hydrocarbon stapled peptide is poorly water-soluble. We have also applied other peptide stapling technology in the further optimization of SP-PROTACs; experiments are still in progress. Other alternatives include nano-based carriers 28, 42. Another disadvantage of SP-PROTACs is the deficient degradation of target proteins, probably because the peptide-based E3 ligase-recruiting motif could be easily cleaved in the cellular environment. A potential solution is to use a small molecule-based E3 ligase-recruiting motif to replace the unstapled peptide-based VHL binding motif.

In this work, the optimized SPMI-HIF2-1 showcased high binding affinity with target proteins, a high helical content, and good proteolytic resistance, attributed to its stapled motif. Notably, SPMI-HIF2-1 efficiently killed HCT116 p53^+/+^ but not p53^-/-^ cancer cells, through an atypical degradation of both MDM2 and MDMX and activation of a p53-dependent apoptotic pathway. Furthermore, SPMI-HIF2-1 exhibited superior therapeutic efficiency in subcutaneous and orthotopic CRC xenograft models and an excellent pharmacokinetic profile. Structural modeling analysis of ternary complexes indicated the binding modes between SPMI-HIF2-1 and MDM2 as well as VHL. In summary, this optimized SP-PROTAC, which promotes degradation of both MDM2 and MDMX, may not only itself be developed into a new class of anticancer agents, but also provide a feasible solution to develop stapled peptides or linear peptide-based PROTACs into drug candidates, especially against enormous pathogenesis-related “undruggable” proteins involved in PPIs.

## Supplementary Material

Supplementary figures.

## Abbreviations

PMI: p53-MDM2/MDMX inhibitor
MDM2: murine double minute 2
MDMX: murine double minute X
PROTAC: proteolysis-targeting chimera
SP: stapled peptide
VHL: Von Hippel Lindau
CRL: Cullin RING E3 ubiquitin ligase
FP: fluorescence polarization
R8: *R*-octenyl-alanine
S5: *S*-pentenyl-alanine
Peg: polyethylene glycol
Fmoc: fluorenylmethyloxycarbonyl
SPPS: solid phase peptide synthesis
RCM: ring closing metathesis
RP-HPLC: reverse-phase high-performance liquid chromatography
HR-MS: high-resolution mass spectrometry
FITC: fluorescein isothiocyanate
CD: circular dichroism
HE: hematoxylin-eosin
TUNEL: TdT-mediated dUTP nick end labeling
IHC: immunohistochemistry
CRC: colorectal cancer
