# Supplementary material for: Design of stapled peptide-based PROTACs for MDM2/MDMX atypical degradation and tumor suppression
Source: Theranostics. 2022 Sep 11;12(15):6665–81. doi: 10.7150/thno.75444 (PMC9516243; doi:10.7150/thno.75444)
Supplement: Supplementary file 1 — Supplementary figures. [file thnov12p6665s1.pdf]

Supplementary Materials

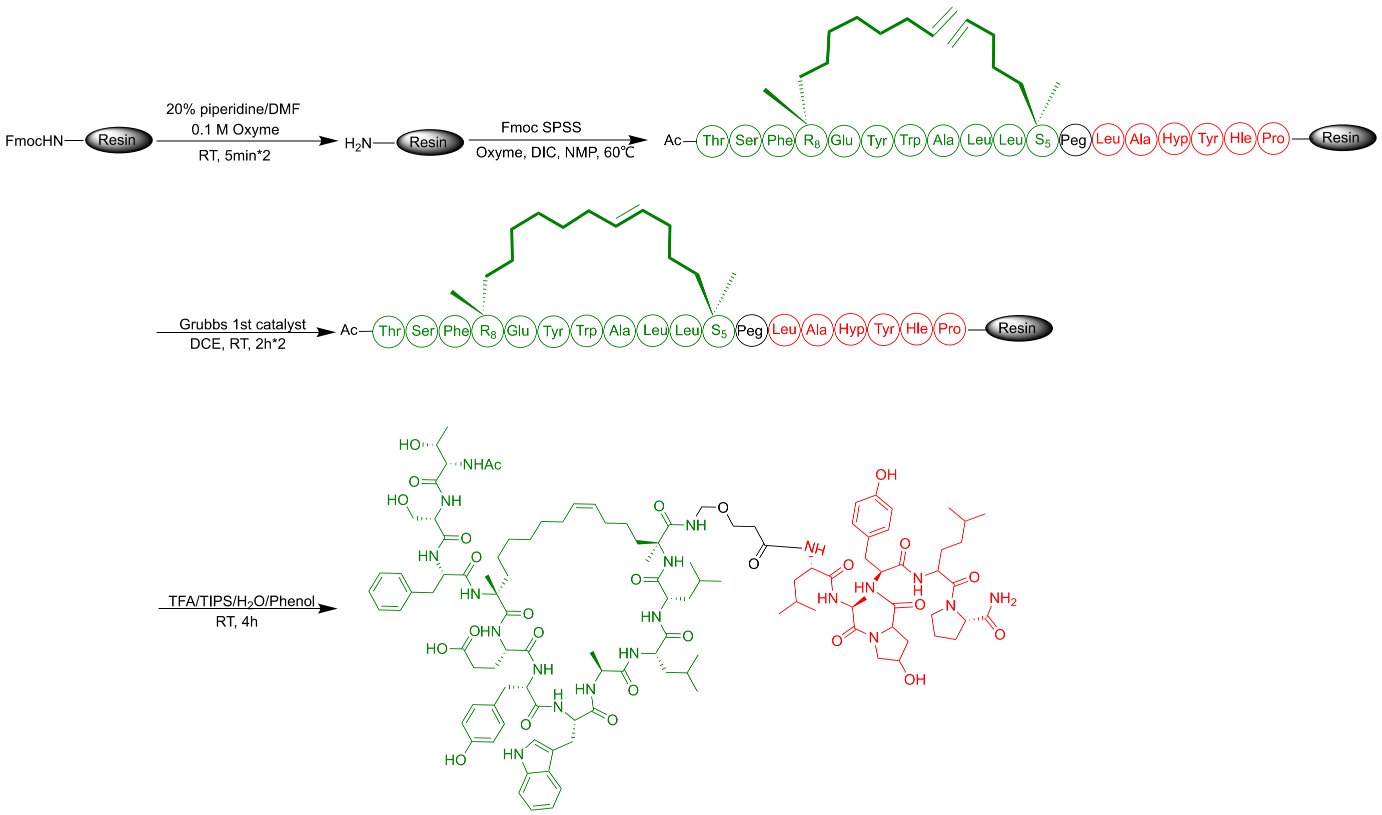

Figure S1. The synthetic route of SP-PROTACs, taken SPMI-HIF2-1 as an example.

Table S1. The yield, chemical formula, and MS data for peptides.

**Peptide**	**Chemical Formula** [M+2H]$^{2+}$	**Calculated Mass** [M+2H]$^{2+}$	**Observed Mass** [M+2H]$^{2+}$
PMI-HIF1-1	$C_{103}H_{148}N_{20}O_{28}^{2+}$	1058.2145	[M+2H]$^{2+}$=1058.0465
PMI-HIF1-2	$C_{106}H_{154}N_{20}O_{29}^{2+}$	1087.0668	[M+2H]$^{2+}$=1087.0668
PMI-HIF1-3	$C_{108}H_{158}N_{20}O_{30}^{2+}$	1109.2810	[M+2H]$^{2+}$=1109.0778
SPMI-HIF1-1	$C_{111}H_{160}N_{20}O_{28}^{2+}$	1112.3065	[M+2H]$^{2+}$=1112.0985
SPMI-HIF1-2	$C_{114}H_{166}N_{20}O_{29}^{2+}$	1141.3465	[M+2H]$^{2+}$=1141.1170
SPMI-HIF1-3	$C_{116}H_{170}N_{20}O_{30}^{2+}$	1163.3730	[M+2H]$^{2+}$=1163.1328
SPMI-HIF2-1	$C_{114}H_{166}N_{20}O_{27}^{2+}$	1125.3475	[M+2H]$^{2+}$=1125.1279
SPMI-HIF2-2	$C_{117}H_{172}N_{20}O_{28}^{2+}$	1154.3875	[M+2H]$^{2+}$=1154.1379
SPMI-HIF2-3	$C_{119}H_{176}N_{20}O_{29}^{2+}$	1176.4140	[M+2H]$^{2+}$=1176.1509
SPMI1	$C_{72}H_{101}N_{13}O_{18}^{2+}$	718.8694	[M+2H]$^{2+}$=718.8770
SPMI2	$C_{75}H_{107}N_{13}O_{17}^{2+}$	732.3775	[M+2H]$^{2+}$=732.3942

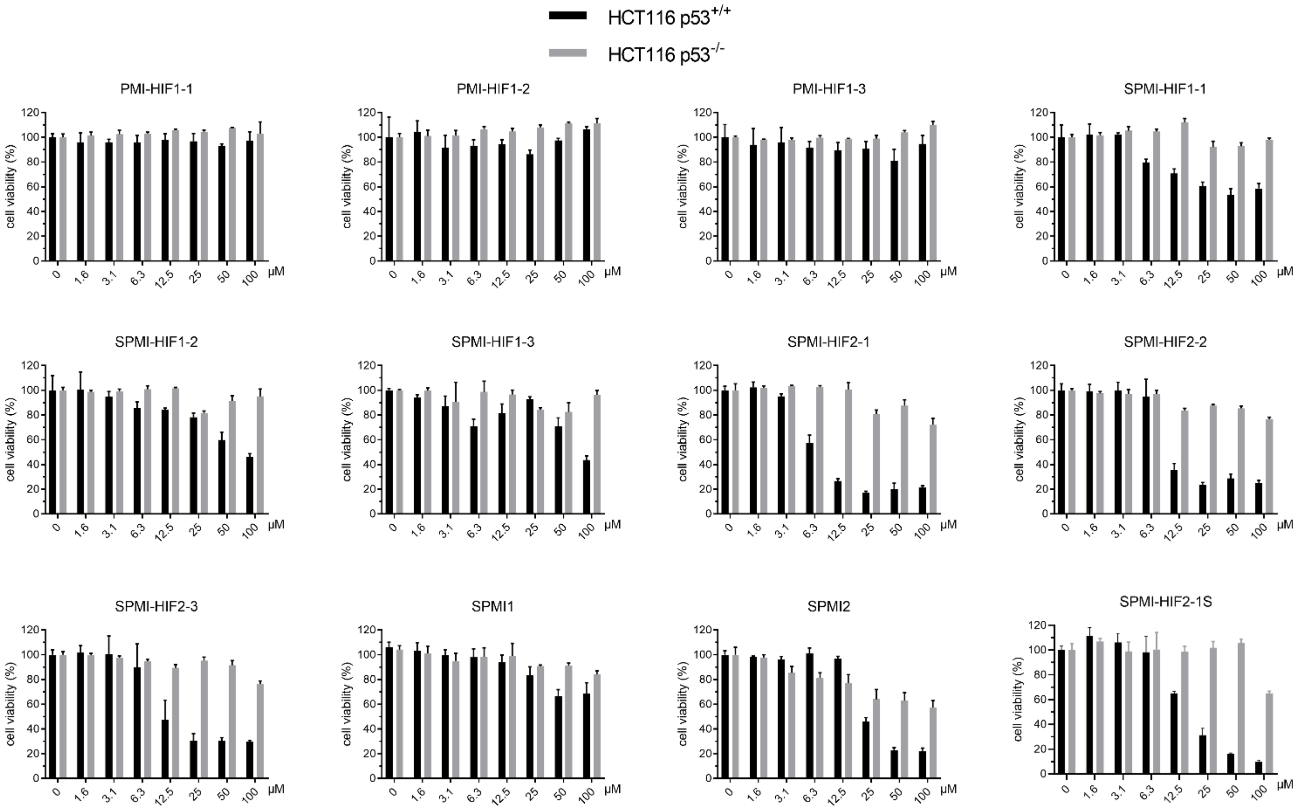

Figure S2. The cell viability of peptides against HCT116 p53$^{+/+}$ and HCT116 p53$^{-/-}$ cancer cells.

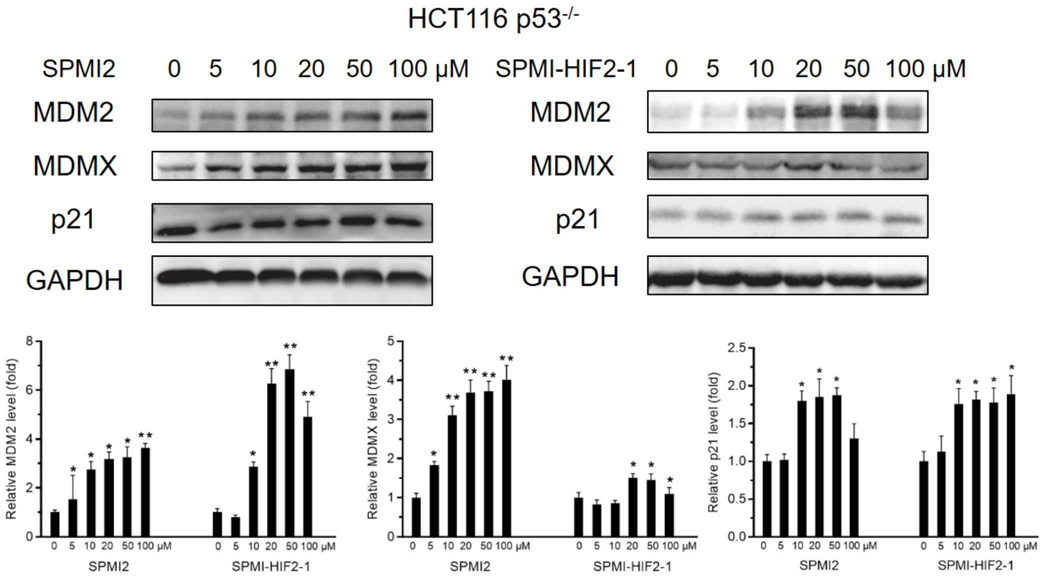

Figure S3. The protein levels of MDM2, MDMX and p21 in HCT116 p53$^{-/-}$ cancer cells treated with SPMI2 and SPMI-HIF2-1 by western blotting assays.

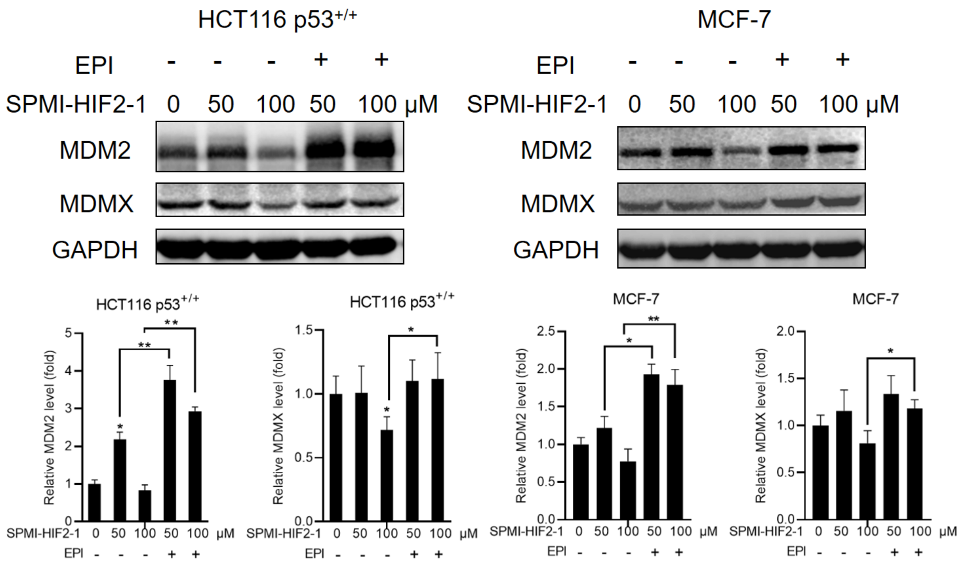

Figure S4. The degradation of MDM2 and MDMX by SPMI-HIF2-1 could be blocked by the addition of 1 μM Epoxomicin (EPI). The protein levels of MDM2 and MDMX were detected by western blotting assays.

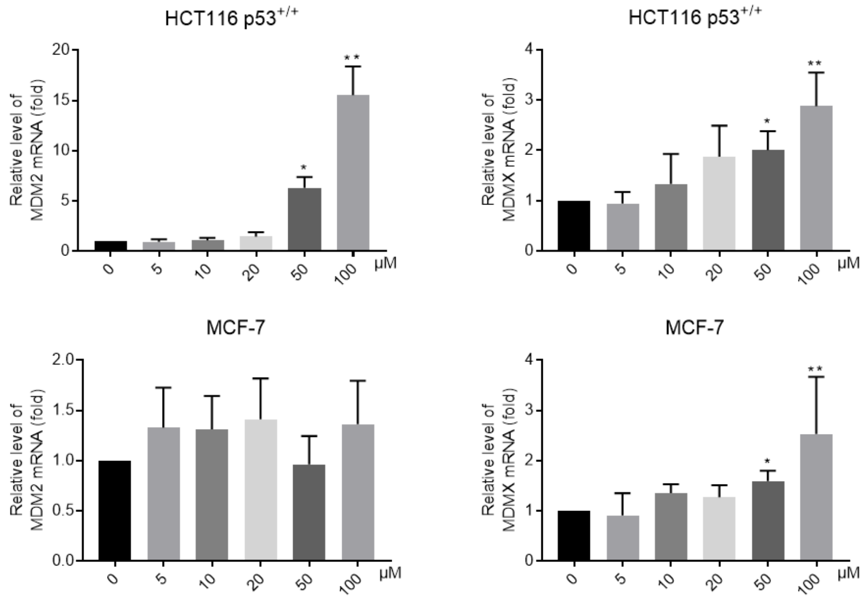

Figure S5. mRNA levels of MDM2 and MDMX treated with SPMI-HIF2-1.

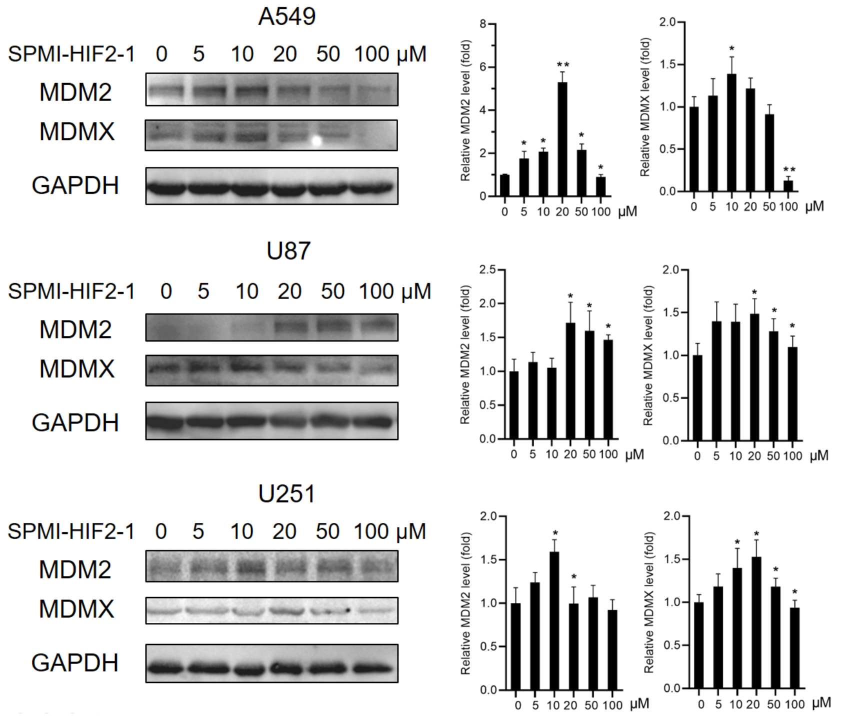

Figure S6. The protein levels of MDM2 and MDMX in various cancer cells treated with SPMI-HIF2-1 by western blotting assays.

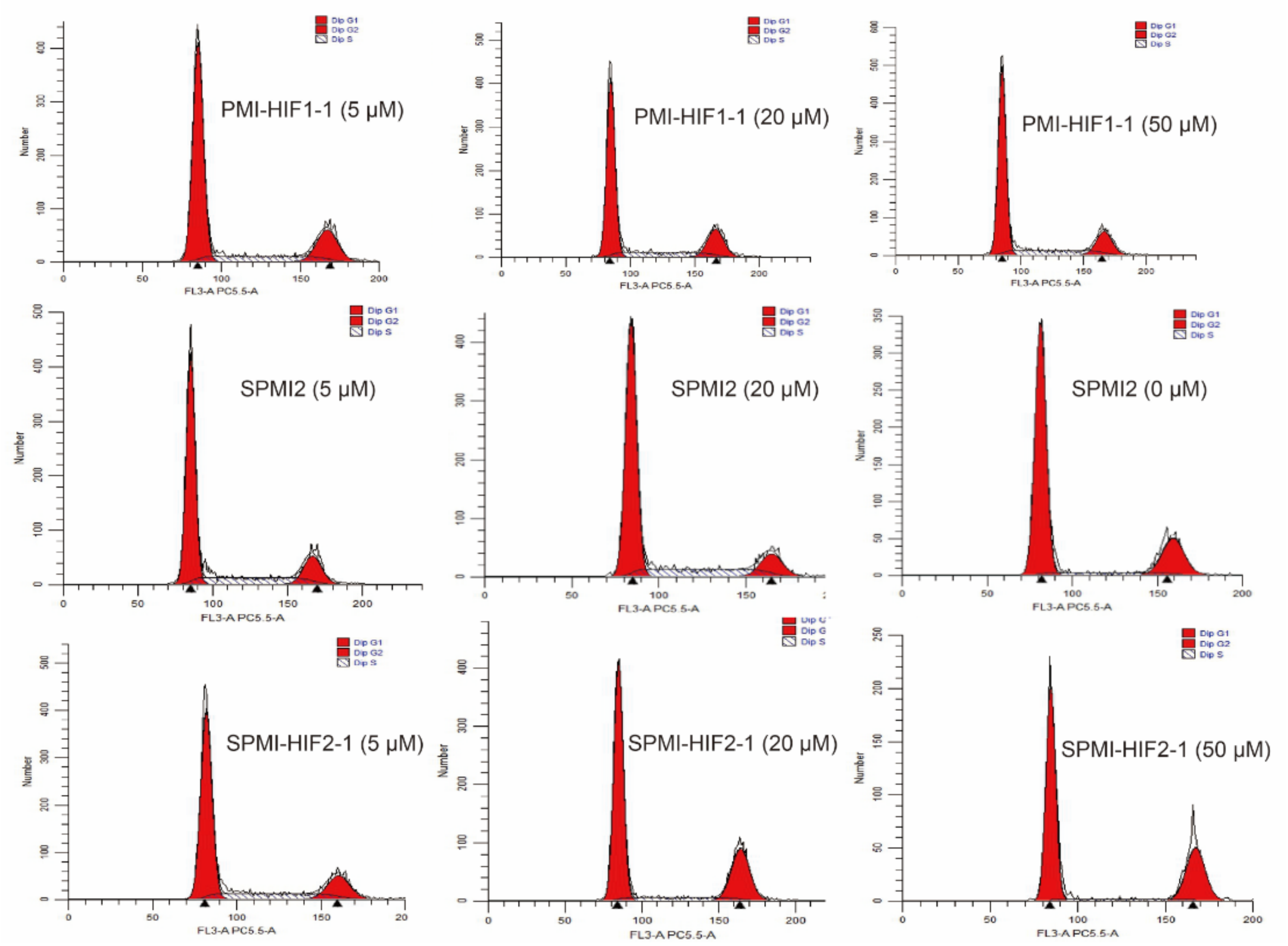

Figure S7. The cell-cycle analysis of PMI-HIF1-1, SPMI2 and SPMI-HIF2-1 in HCT116 p53$^{+/+}$ cancer cells.

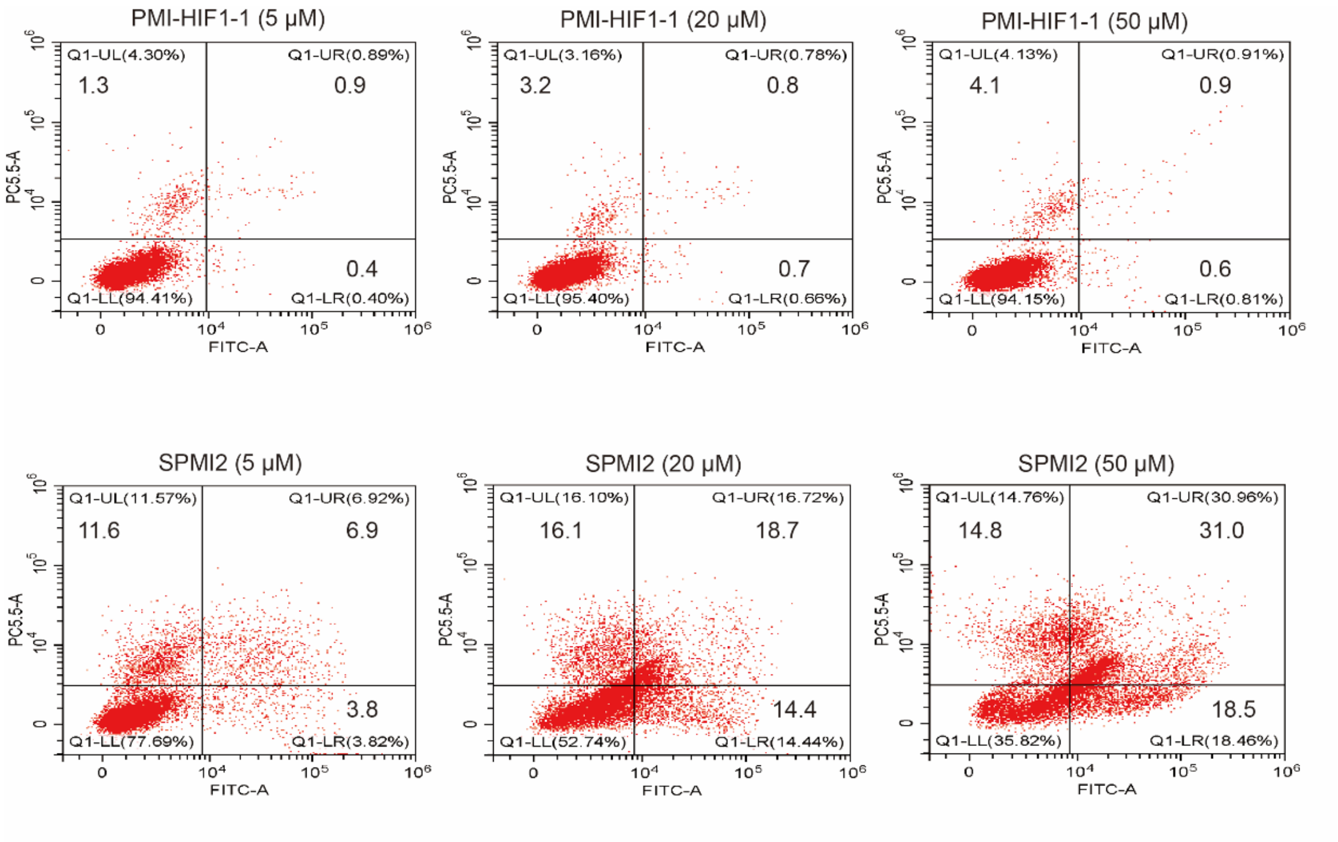

Figure S8. The apoptotic analysis of PMI-HIF1-1 and SPMI2 in HCT116 p53$^{+/+}$ cancer cells.

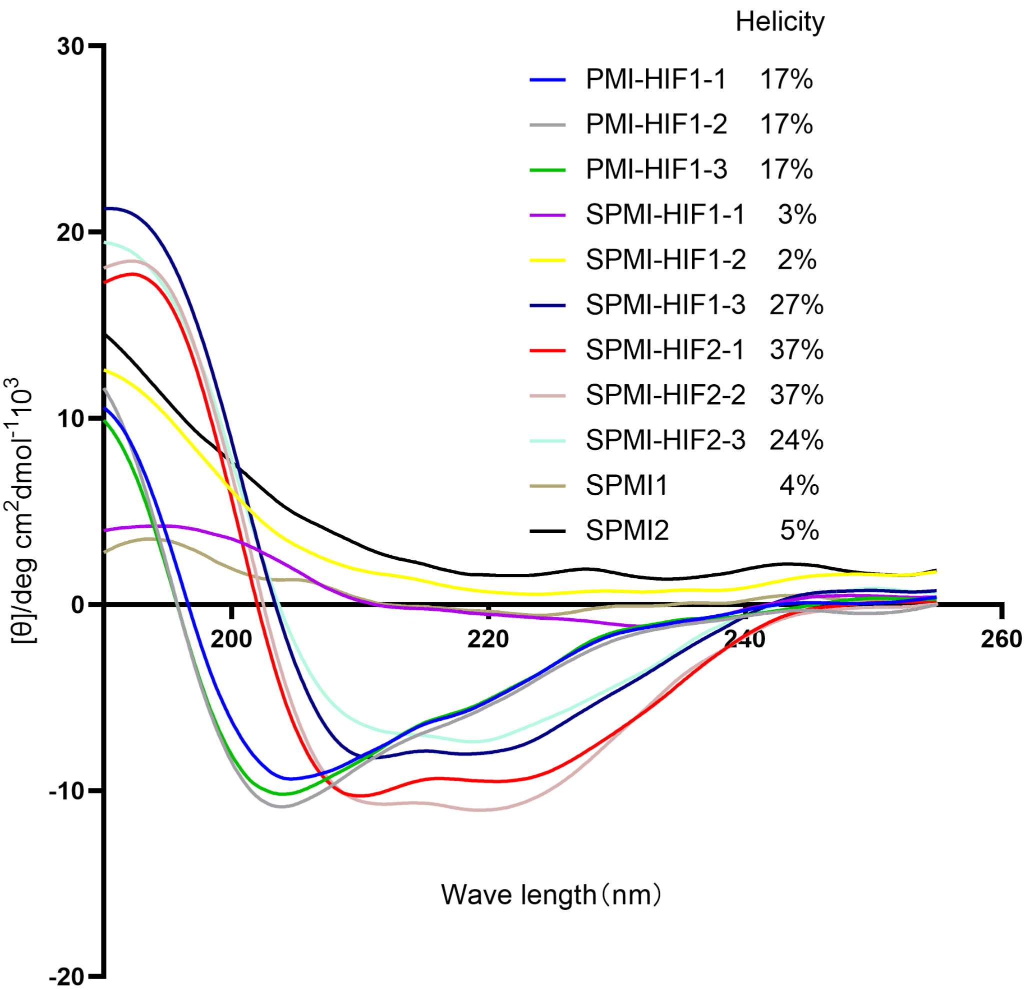

Figure S9. CD spectra of peptides in this study.

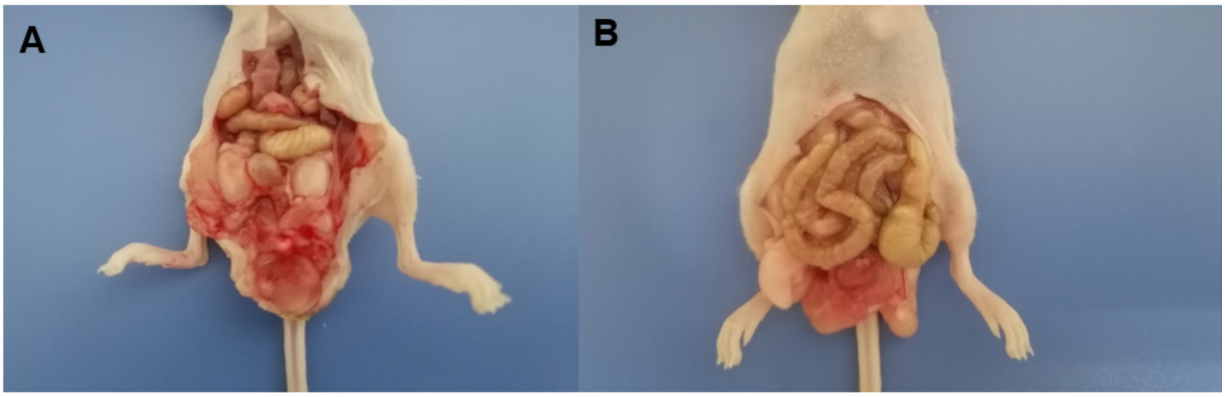

Figure S10. CRC xenograft models through the orthotopical implantation of HCT116 cells. **A** showed the representative mice image bearing orthotopical tumor and **B** showed the negative control.

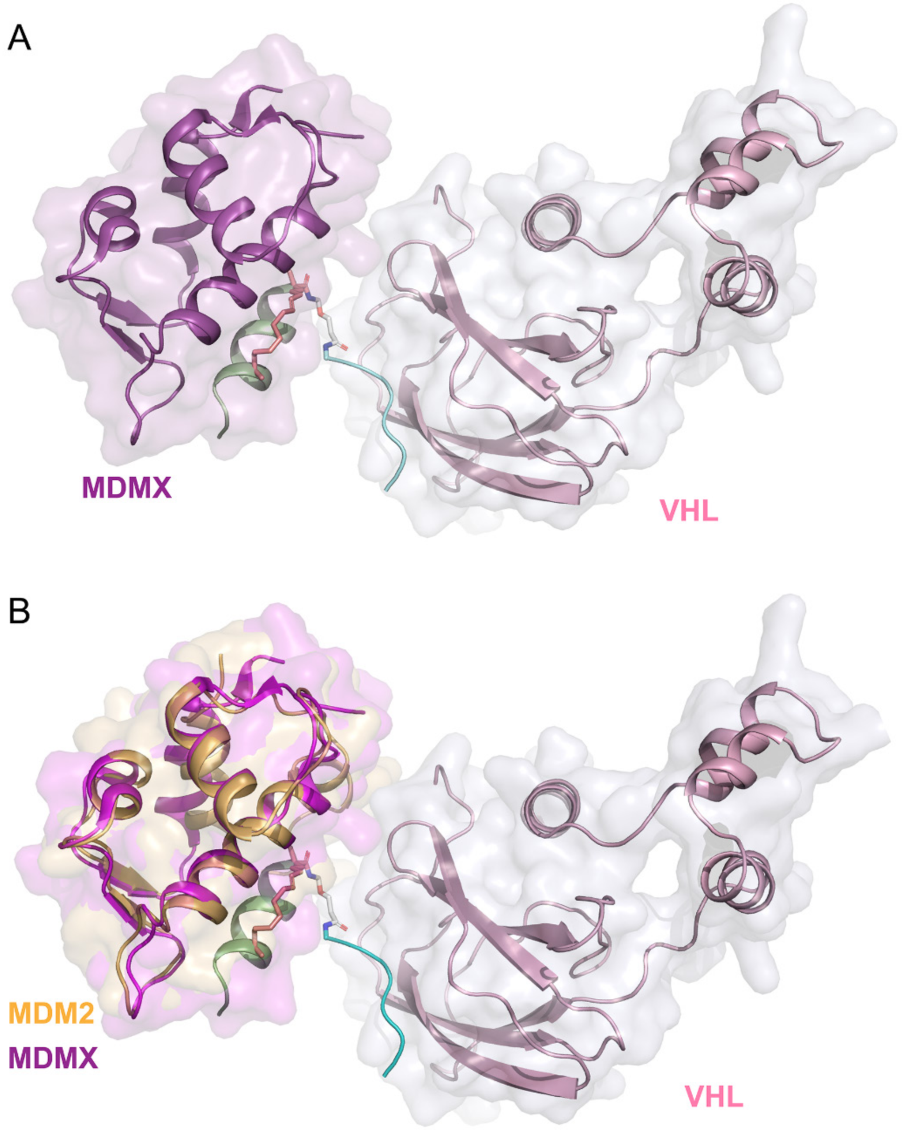

Figure S11. Structural modeling analysis of ternary complex between SPMI-HIF2-1, MDMX and VHL. (A) The overview of SPMI-HIF2-1 complexed with target protein MDMX and E3 ligase VHL. MDMX binding motif was shown as palegreen cartoon and hydrocarbon staple was shown as salmon sticks. Peg linker was shown as white sticks and VHL binding motif was shown as cyan coil. **(B)** A structural comparison between MDM2/MDMX complexes. MDM2 was shown as wheat cartoon and MDMX magenta cartoon.

Characterization of SP-PROTACs by HPLC and HR-MS.

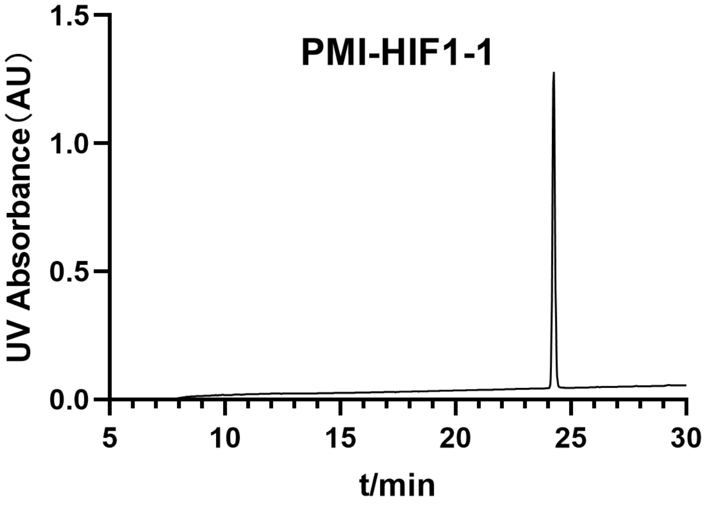

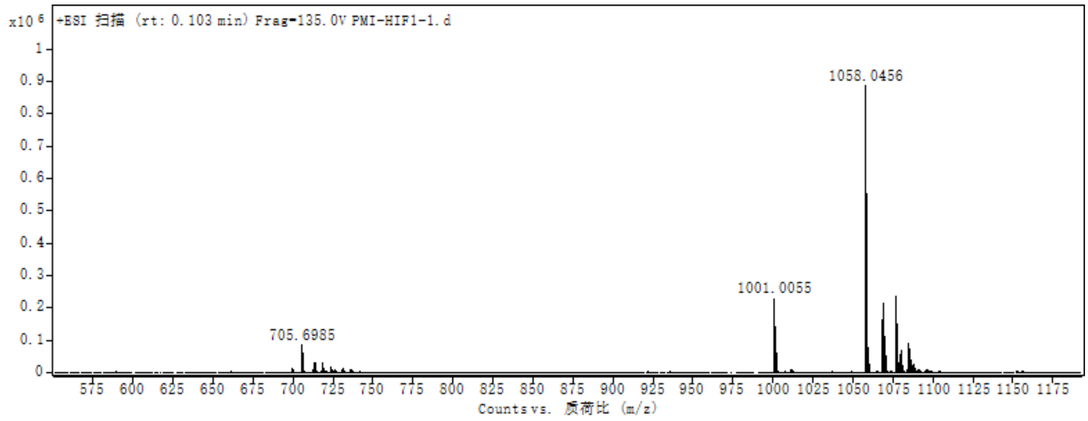

HR-Q-TOF-MS and HPLC of compound PMI-HIF1-1.

PMI-HIF1-1: HR-Q-TOF-MS *m/z* calcd for $C_{103}H_{148}N_{20}O_{28}$ 2114.4290; found $[M+2H]^{2+}$=1058.0456,$[M+3H]^{3+}$=705.6985.

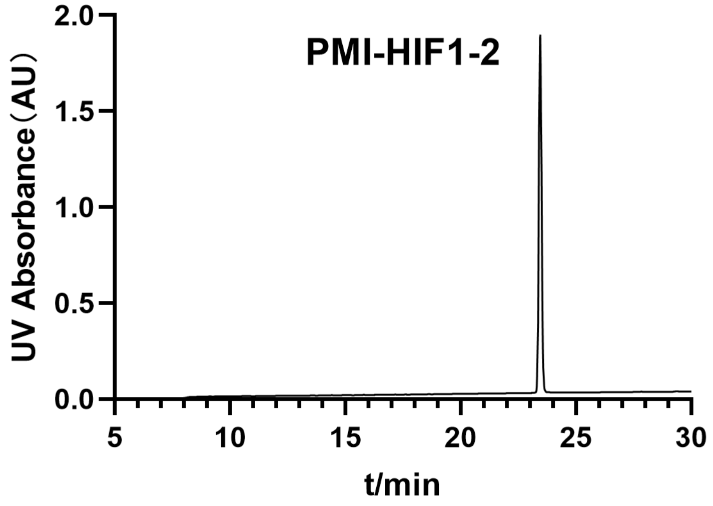

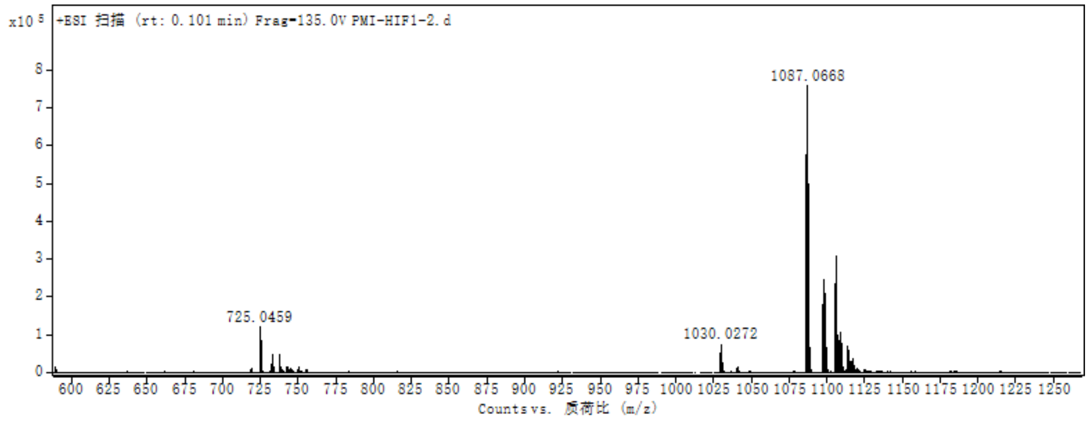

HR-Q-TOF-MS and HPLC of compound PMI-HIF1-2.

PMI-HIF1-2: HR-Q-TOF-MS *m/z* calcd for $C_{106}H_{154}N_{20}O_{29}$ 2172.5090; found $[M+2H]^{2+}$=1087.0668, $[M+3H]^{3+}$=725.0459.

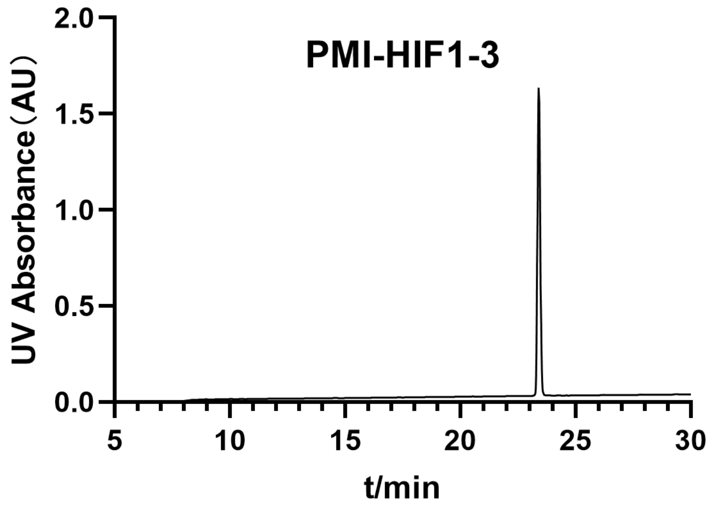

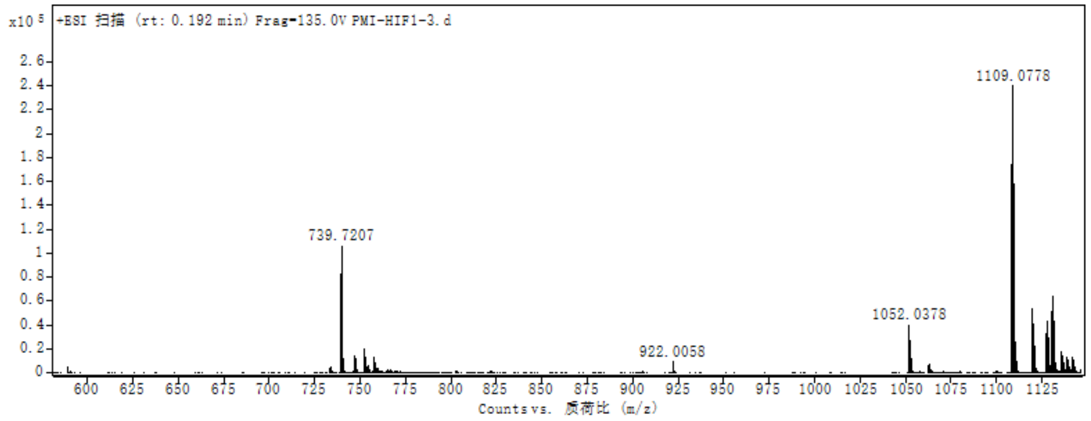

HR-Q-TOF-MS and HPLC of compound PMI-HIF1-3.

PMI-HIF1-3: HR-Q-TOF-MS *m/z* calcd for $C_{108}H_{158}N_{20}O_{30}$ 2216.5620; found $[M+2H]^{2+}$=1109.0778, $[M+3H]^{3+}$=739.7207.

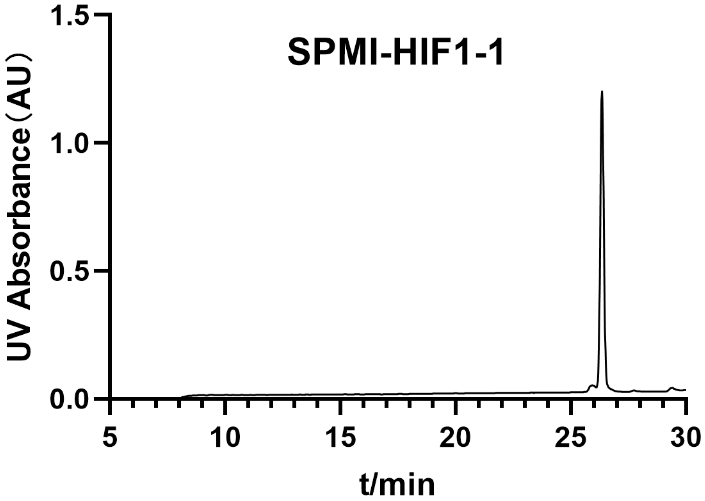

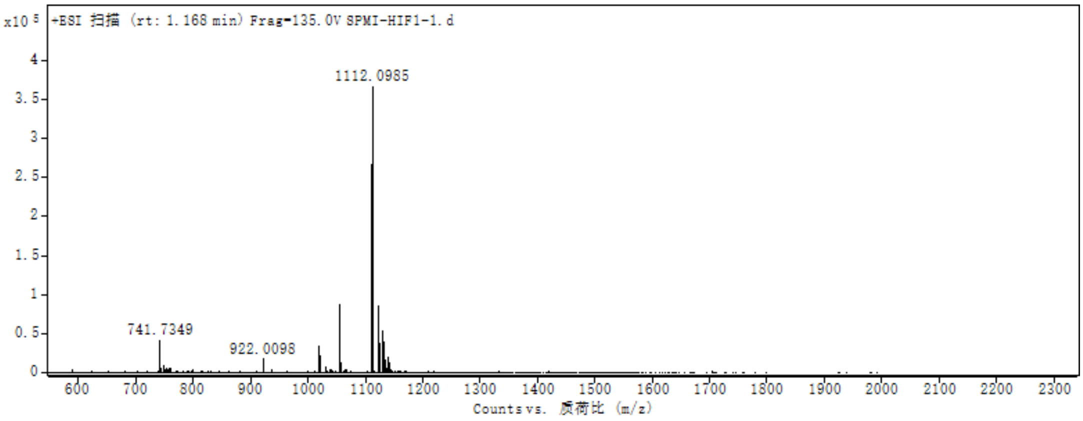

HR-Q-TOF-MS and HPLC of compound SPMI-HIF1-1.

SPMI-HIF1-1: HR-Q-TOF-MS *m/z* calcd for $C_{111}H_{160}N_{20}O_{28}$ 2222.6130; found $[M+2H]^{2+}$=1112.0985, $[M+3H]^{3+}$=741.7349.

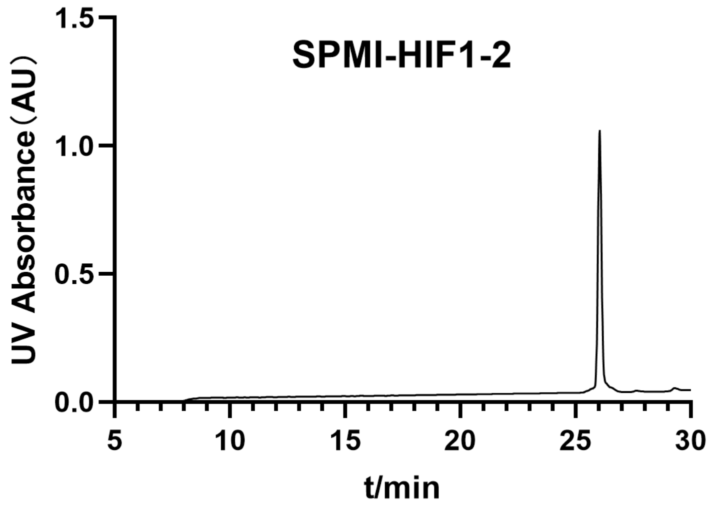

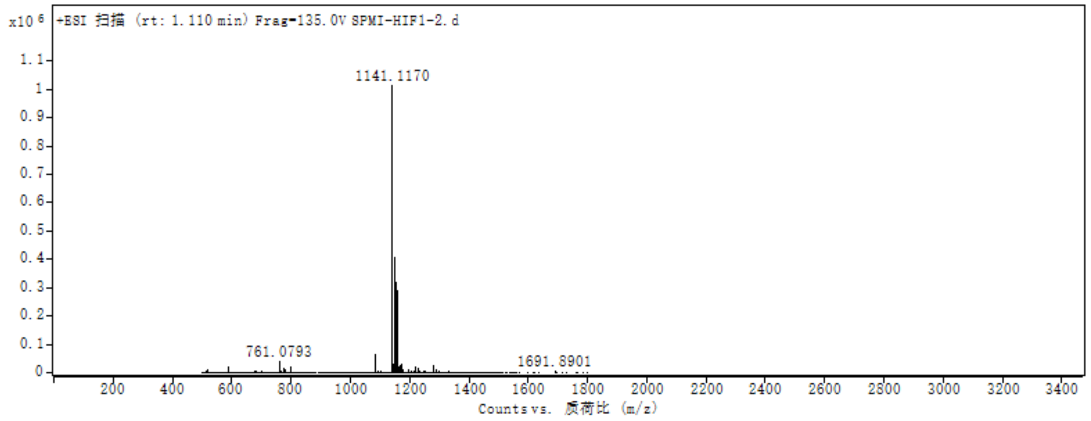

HR-Q-TOF-MS and HPLC of compound SPMI-HIF1-2.

SPMI-HIF1-2: HR-Q-TOF-MS *m/z* calcd for $C_{114}H_{166}N_{20}O_{29}$ 2280.6930; found $[M+2H]^{2+}$=1141.1170, $[M+3H]^{3+}$=761.0793.

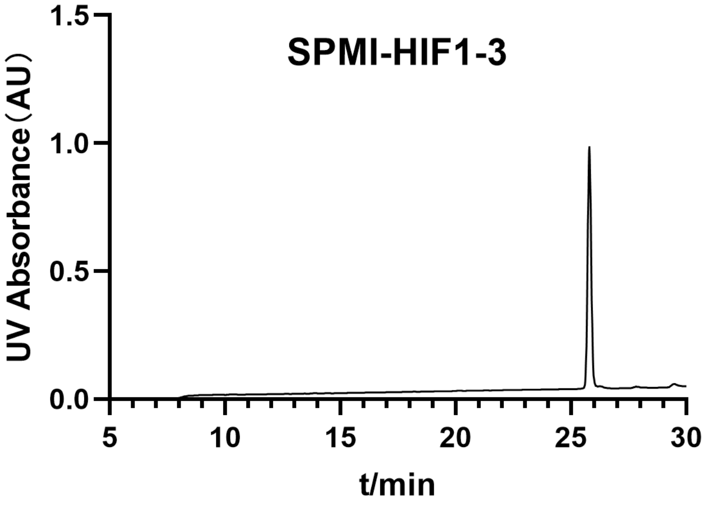

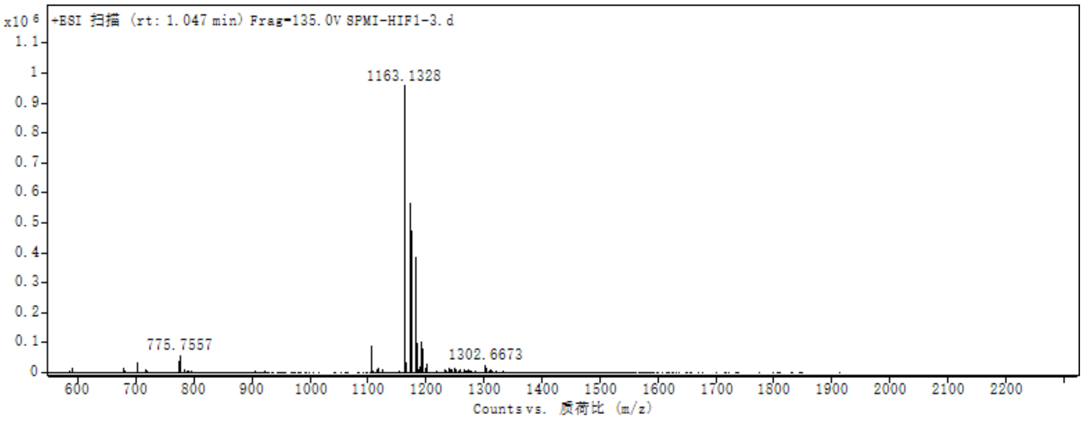

HR-Q-TOF-MS and HPLC of compound SPMI-HIF1-3.

SPMI-HIF1-3: HR-Q-TOF-MS *m/z* calcd for $C_{116}H_{170}N_{20}O_{30}$ 2324.7460; found $[M+2H]^{2+}$=1163.1328, $[M+3H]^{3+}$=775.7557.

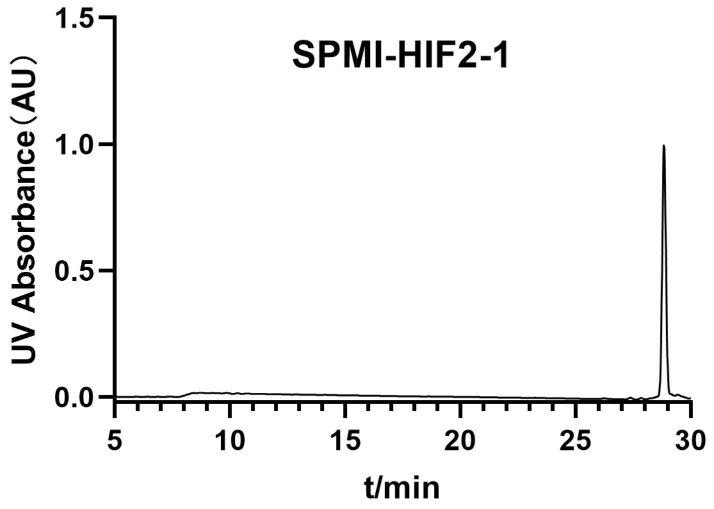

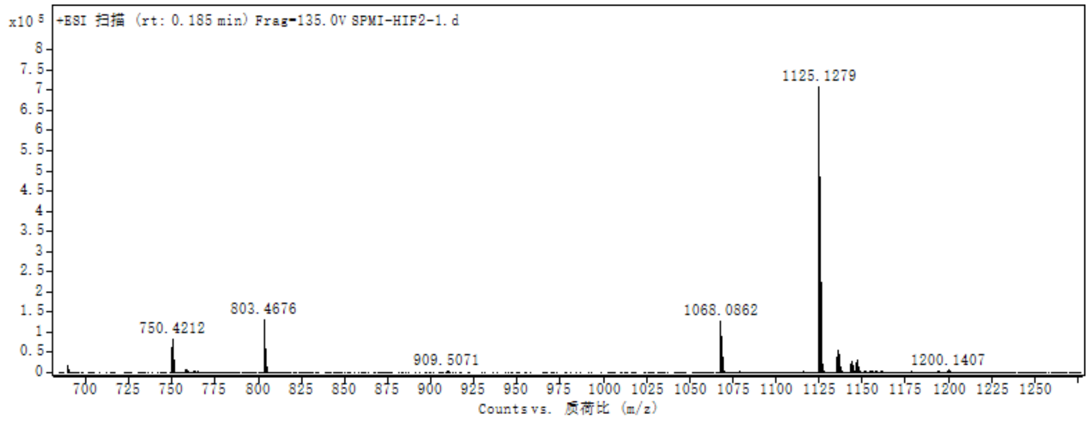

HR-Q-TOF-MS and HPLC of compound SPMI-HIF2-1.

SPMI-HIF2-1: HR-Q-TOF-MS *m/z* calcd for $C_{114}H_{166}N_{20}O_{27}$ 2248.6950; found $[M+2H]^{2+}$=1125.1279, $[M+3H]^{3+}$=750.4212.

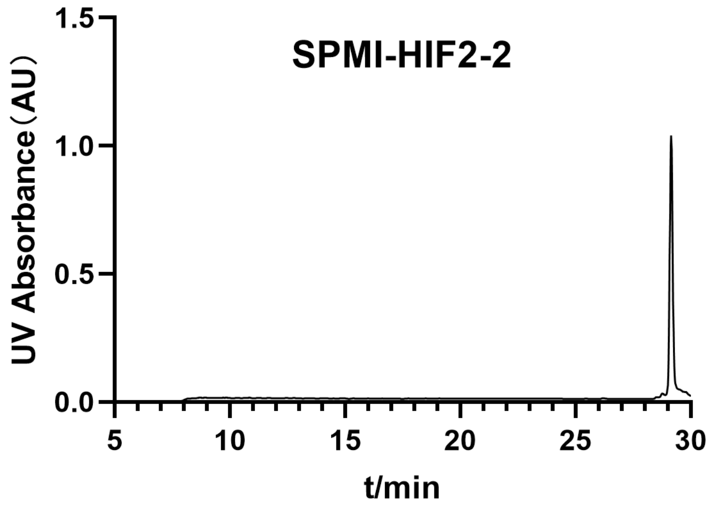

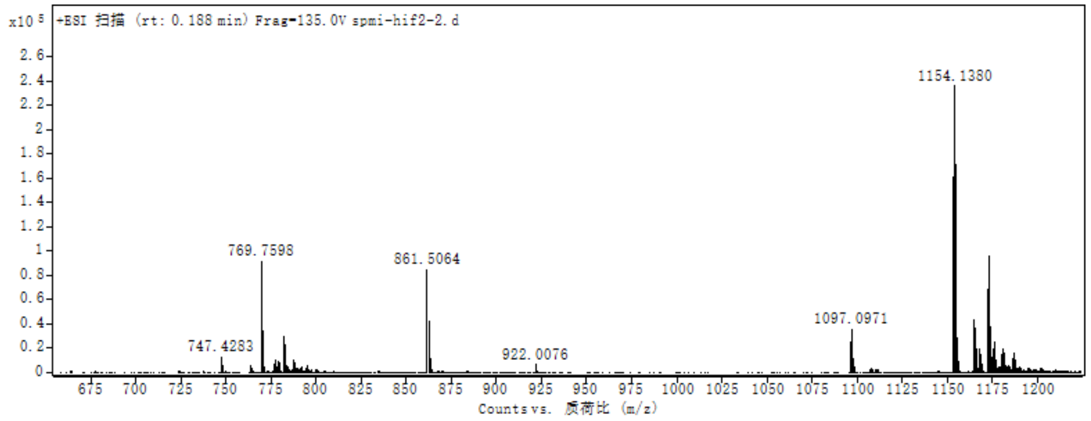

HR-Q-TOF-MS and HPLC of compound SPMI-HIF2-2.

SPMI-HIF2-2: HR-Q-TOF-MS *m/z* calcd for $C_{117}H_{172}N_{20}O_{28}$ 2306.7750; found $[M+2H]^{2+}$=1154.1380, $[M+3H]^{3+}$=769.7598.

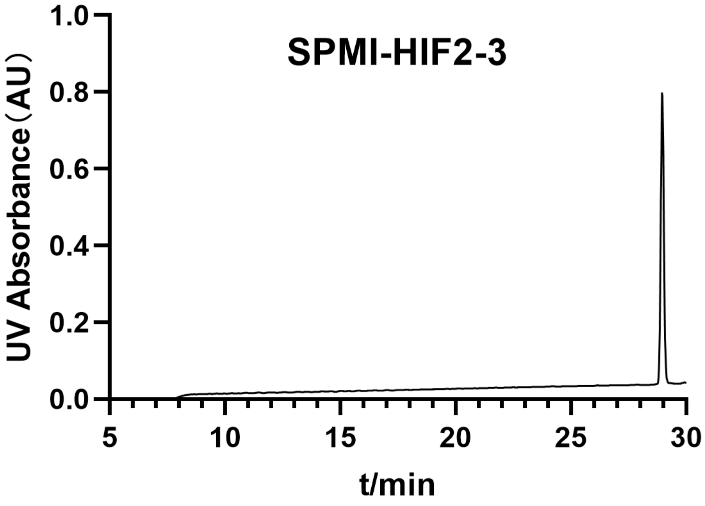

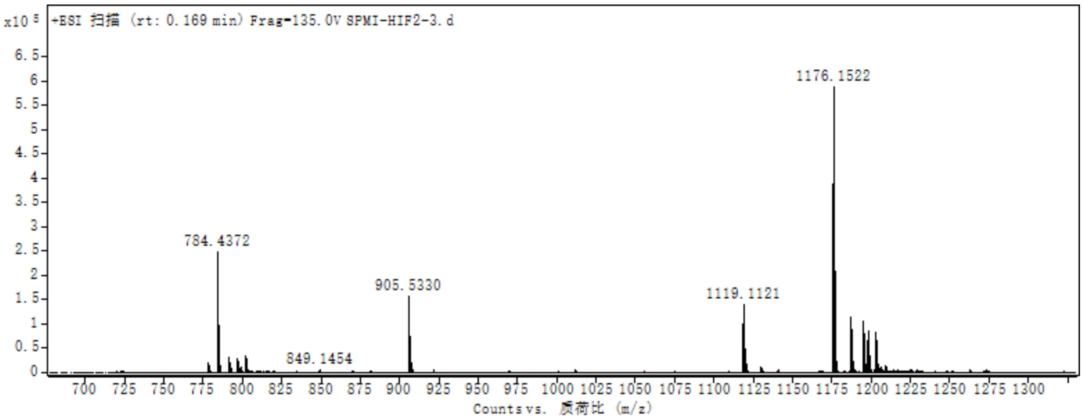

HR-Q-TOF-MS and HPLC of compound SPMI-HIF2-3.

SPMI-HIF2-3: HR-Q-TOF-MS *m/z* calcd for $C_{119}H_{176}N_{20}O_{29}$ 2250.8280; found $[M+2H]^{2+}$=1176.1522, $[M+3H]^{3+}$=784.4372.

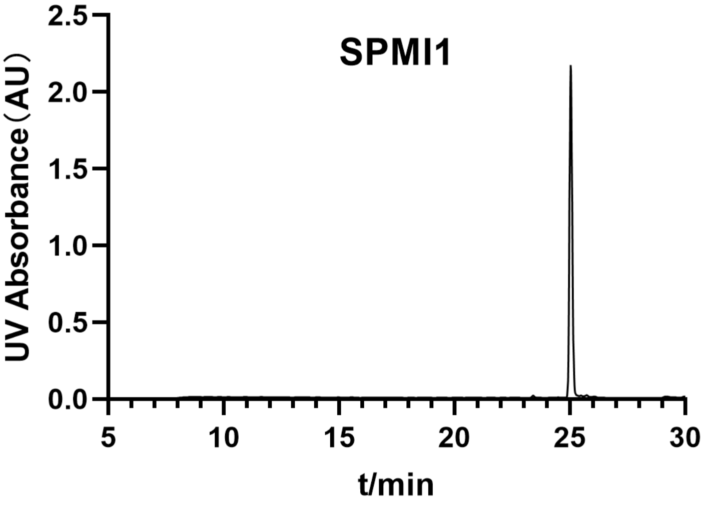

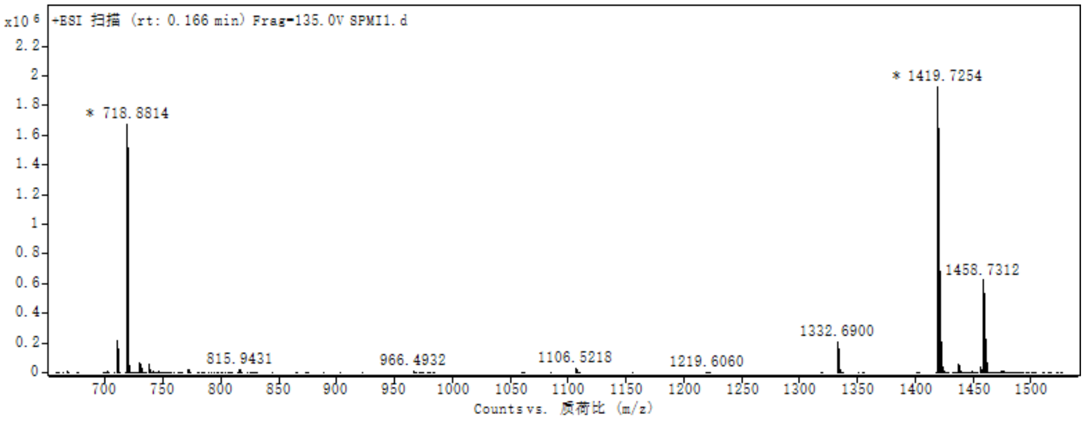

HR-Q-TOF-MS and HPLC of compound SPMI1.

SPMI1: HR-Q-TOF-MS *m/z* calcd for $C_{72}H_{101}N_{13}O_{18}$ 1435.7388; found $[M+Na]^{+}$=1458.7312, $[M+2H]^{2+}$=718.8814.

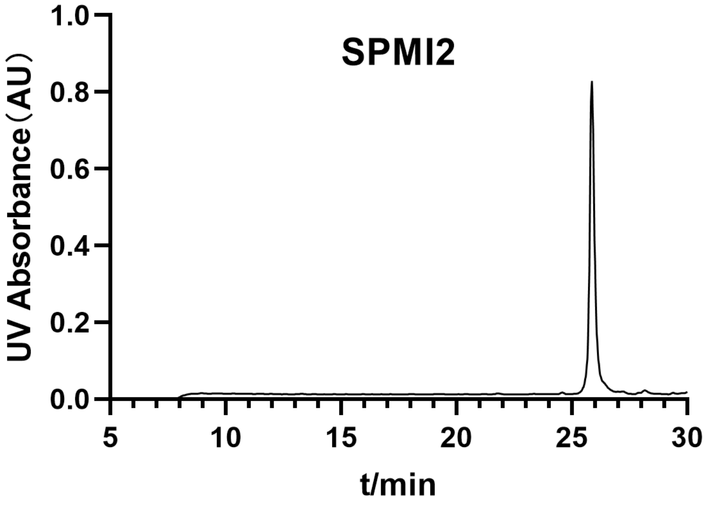

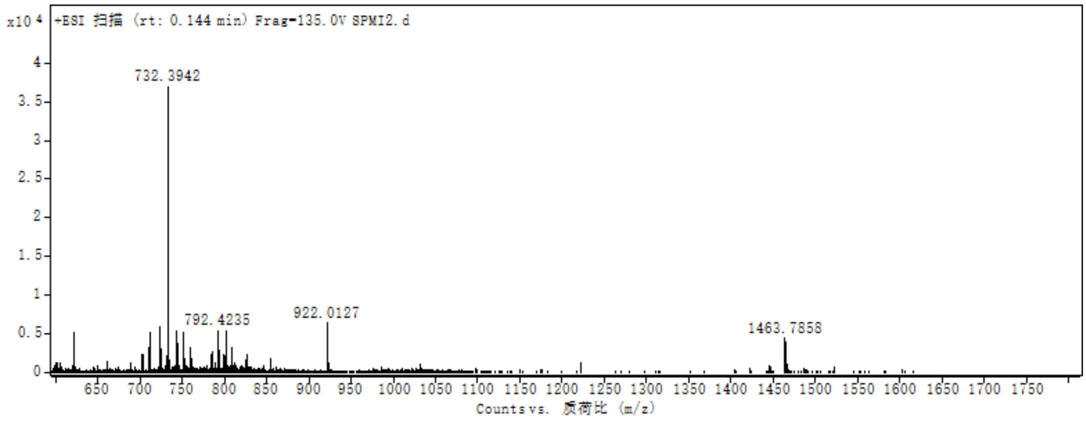

HR-Q-TOF-MS and HPLC of compound SPMI2.

SPMI2: HR-Q-TOF-MS *m/z* calcd for $C_{72}H_{101}N_{13}O_{18}$ 1462.755; found $[M+H]^{+}$=1463.7858, $[M+2H]^{2+}$=732.3942.
